# Uncovering the rapidly evolving orbits of the dynamic TOI-201 system

**DOI:** 10.1126/sciadv.aef2618

**Published:** 2026-04-15

**Authors:** Ismael Mireles, Solène Ulmer-Moll, Donald Liveoak, Diana Dragomir, Judith Korth, Alexander Venner, Karen A. Collins, Amaury H.M.J. Triaud, Tristan Guillot, Antoine Petit, Theron Carmichael, Sarah Millholland, Tim Hallatt, Hannu Parviainen, Hugh P. Osborn, David Rapetti, Thomas A. Baycroft, Siddharth Bhatnagar, François Bouchy, Radmila Dancikova, Pedro Figueira, Monika Lendl, Stéphane Udry, Peter Wheatley, Lyu Abe, Abdelkrim Agabi, Matteo Beltrame, Philippe Bendjoya, Vincent Deloupy, Djamel Mékarnia, François-Xavier Schmider, Olga Suárez, Khalid Barkaoui, Keith Horne, Felipe Murgas, Enric Palle, Richard P. Schwarz, Ramotholo Sefako, Avi Shporer, Gregor Srdoc, Chris Stockdale, Francis P. Wilkin, Joel D. Hartman, Lauren A. Sgro, Thiam-Guan Tan, Jon M. Jenkins, Attila Bódi, David Havell, Darren Rivett, Ian Transom

**Affiliations:** ^1^ Department of Physics and Astronomy, University of New Mexico, Albuquerque, NM 87106, USA.; ^2^ Leiden Observatory, Leiden University, Leiden, Netherlands.; ^3^ Department of Physics and Kavli Institute for Astrophysics and Space Research, Massachusetts Institute of Technology, Cambridge, MA 02139, USA.; ^4^ MIT Kavli Institute for Astrophysics and Space Research, Massachusetts Institute of Technology, Cambridge, MA 02139, USA.; ^5^ Department of Physics, University of Michigan, Ann Arbor, MI 48109, USA.; ^6^ Observatoire astronomique de l’Université de Genève, Versoix 1290, Switzerland.; ^7^ Centre for Astrophysics, University of Southern Queensland, Toowoomba, QLD 4350, Australia.; ^8^ Max Planck Institute for Astronomy, 69117 Heidelberg, Germany.; ^9^ Center for Astrophysics, Harvard & Smithsonian, Cambridge, MA 02138, USA.; ^10^ School of Physics & Astronomy, University of Birmingham, Edgbaston, Birmingham B15 2TT, UK.; ^11^ Observatoire de la Côte d’Azur, UniCA, Laboratoire Lagrange, CNRS UMR 7293, Nice Cedex 4, France.; ^12^ Institute for Astronomy, University of Hawai’i, Honolulu, HI 96822, USA.; ^13^ Departamento de Astrofísica, Universidad de La Laguna (ULL), La Laguna E-38206, Spain.; ^14^ Instituto de Astrofísica de Canarias (IAC), La Laguna E-38200, Spain.; ^15^ NCCR/Planet-S, Physikalisches Institut, Universität Bern, Bern, Switzerland.; ^16^ Institut für Teilchen- und Astrophysik, ETH Zürich, Zürich, Switzerland.; ^17^ NASA Ames Research Center, Moffett Field, CA 94035, USA.; ^18^ Research Institute for Advanced Computer Science, Universities Space Research Association, Washington, DC 20024, USA.; ^19^ Tsung-Dao Lee Institute, Shanghai Jiao Tong University, 1 Lisuo Road, Shanghai 201210, China.; ^20^ Group of Applied Physics and Institute for Environmental Sciences, Université de Genève, Genéve, Switzerland.; ^21^ Institute of Physics, École Polytechnique Fédérale de Lausanne (EPFL), Observatoire de Sauverny, Versoix, Switzerland.; ^22^ Instituto de Astrofísica de Andalucía-CSIC, Glorieta de la Astronomía s/n, E-18008 Granada, Spain.; ^23^ Department of Physics, University of Warwick, Coventry CV4 7AL, UK.; ^24^ PNRA & IPEV, Concordia Station, Antarctica.; ^25^ Astrobiology Research Unit, Université de Liège, Liège, Belgium.; ^26^ Department of Earth, Atmospheric and Planetary Science, Massachusetts Institute of Technology, Cambridge, MA 02139, USA.; ^27^ SUPA Physics and Astronomy, University of St. Andrews, Fife, KY16 9SS Scotland, UK.; ^28^ South African Astronomical Observatory, P.O. Box 9, Observatory, Cape Town 7935, South Africa.; ^29^ Kotizarovci Observatory, Viskovo, Croatia.; ^30^ Hazelwood Observatory, Hazelwood, Australia.; ^31^ Department of Physics and Astronomy, Union College, Schenectady, NY 12308, USA.; ^32^ Department of Astrophysical Sciences, Princeton University, Princeton, NJ 08544, USA.; ^33^ SETI Institute, Carl Sagan Center, Mountain View, CA 94043, USA.; ^34^ Perth Exoplanet Survey Telescope, Perth, Western Australia, Australia.; ^35^ SETI Institute & Unistellar Citizen Science Network, Pukorokoro, Thames-Coromandel, New Zealand.; ^36^ SETI Institute & Unistellar Citizen Science Network, Lake Macquarie, New South Wales, Australia.; ^37^ SETI Institute & Unistellar Citizen Science Network, Cambridge Waikato, New Zealand.

## Abstract

Studying planetary interactions in exoplanet systems informs theories of planet formation and evolution, providing essential context for understanding our own solar system. We combine spectroscopy, transit photometry, transit timing variations, and astrometry to characterize the TOI-201 system. The cotransiting system consists of a super-Earth, warm Jupiter, and massive companion at 5.8-, 53-, and 2900-day orbital periods, respectively. We perform dynamical simulations to study the past and future of the system. von-Zeipel-Kozai-Lidov oscillations emerge as the most plausible scenario to explain the outer companion’s high orbital eccentricity, with planet-planet scattering a possible but less likely contender. Because of nonzero mutual inclinations between the planets, the system is visibly evolving on very short timescales, with the current cotransiting configuration ending in 200 years.

## Teaser

A rare cotransiting system with a super-Earth, warm Jupiter, and brown dwarf is visibly evolving on human-observable timescales.

## INTRODUCTION

Most of the giant exoplanets discovered to date have properties that are, for the most part, very different from those of the Solar System gas giants. They tend to orbit much closer to their host star, and are often found in noncircular orbits. When those giant exoplanets reside in multiplanet systems, a wealth of new clues regarding their dynamical evolution becomes available.

The Transiting Exoplanet Survey Satellite, or TESS, has discovered over 650 new planets, 242 of which are part of multiplanet systems. Unlike the previous transit surveys, TESS is an all-sky survey observing stars of all brightness instead of focusing on fainter stars or specific regions of the sky. As a result, TESS has discovered planets around bright stars that are ideal for follow-up observations to characterize the planets in unprecedented detail. One such planet discovered by TESS is TOI-201 b, a warm Jupiter orbiting a relatively bright F-type star at a 53 day period (*1*). Warm Jupiters are defined as giant planets with orbital periods between 10 and 200 days. Often described as bridging the gap between hot Jupiters and Jovian analogs, these planets have been subjected to numerous studies over the past decade (*2*–*4*).

We are beginning to understand how these planets interact with other planets in the same system. Studies of warm Jupiters observed by Kepler have found that the majority of them have small, nearby companions (*3*, *4*). These results suggest that warm Jupiter systems likely formed in situ (*5*) or further out beyond the ice line before undergoing disk-driven migration (*6*, *7*), as these mechanisms tend to preserve nearby planets. This is in contrast to hot Jupiters, whose general lack of nearby companions point to more dynamically violent mechanisms as the origin of those systems (*8*–*10*). While warm Jupiters are more likely to have nearby small companions than hot Jupiters, the opposite is true for massive, distant companions. Studies have found that hot Jupiters are more likely to have massive (between 1 and 20 Jupiter masses), distant (between 1 and 20 AU) companions than warm Jupiters, although the difference in their occurrence rate is not as substantial as the difference in occurrence rate of small planets (*11*). Nonetheless, this difference again suggests distinct mechanisms at play for hot and warm Jupiter systems, as massive, distant companions drive the more dynamically active pathways (*12*). Finding systems with both nearby and distant companions will provide a more complete picture of warm Jupiter formation pathways.

Among warm Jupiters with outer companions, there is a small but growing sample of systems in which the companions have masses close to the brown dwarf lower mass limit. These systems open the door to potential secular interactions that could be probed observationally on timescales as short as a human lifetime.

## RESULTS

### Identifying two new companions

The TESS mission originally identified the warm Jupiter TOI-201 b as a candidate on 7 May 2019 (*13*). It was later confirmed as a planet with a mass of 0.42 Jupiter masses and a moderately eccentric orbit (*e* ~ 0.28) (*1*). TESS later identified a second candidate in the system, TOI-201 d, on 11 March 2020, a potential super-Earth with an orbital period of 5.85 days. While this candidate was known at the time of the confirmation of the warm Jupiter, it was not confirmed, as its radial velocity (RV) signal was too weak to be detected. This is partly due to a linear trend seen in the RVs that was attributed solely to stellar activity. However, as we describe here, the trend was due to an additional massive planet exterior to the warm Jupiter. Here, we statistically validate TOI-201 d using the triceratops package (*14*) and obtain a tentative mass measurement.

We visually identified a single, partial transit event in TESS Sector 64 that was unrelated to the super-Earth and warm Jupiter and corresponded to the recently confirmed TOI-201 c (*15*). The transit coincided with variations in the timing of the transits of the warm Jupiter, with the transits immediately after the single transit occurring about 30 min later than expected. The fact that these sudden transit timing variations (TTVs) occurred so close to the single transit suggested that whatever caused the single transit was also responsible for TOI-201 b’s TTVs.

### Determining the orbital period of the outer companion

Having transited only once and partially during TESS observations, we had minimal constraints on the orbital period of the outer companion. However, we used the duration of the transit to obtain a rough estimate of the orbital period. Assuming a circular orbit, the 13-hour transit duration implied an orbital period of approximately 250 days (*16*). To motivate our follow-up observation strategy, we used the TESS data to determine where additional transits could have fallen into data gaps. The shortest possible orbital period was 200 days and there were many possible periods below 500 days that could be observed using ground-based photometry. We observed some from the ground using a variety of telescopes across the world to try to detect a second transit. The telescopes were those from the Las Cumbres Observatory Global Telescope Network (LCOGT), Perth Exoplanet Survey Telescope (PEST), Hazelwood Observatory, HATPI, and Unistellar Network. At the same time, we monitored the system using RV measurements from the CORALIE spectrograph on the Swiss 1.2-m Leonhard Euler Telescope and HARPS spectrograph on the European Southern Observatory’s (ESO) 3.6-m telescope, both at ESO La Silla Observatory in Chile, as well as Carnegie’s Planet Finder Spectrograph (PFS) attached to the 6.5-m Magellan Clay telescope at Las Campanas Observatory in Chile. The photometric observations revealed no new transits while the RVs showed a 200 m/s drop nearly 4 years after the RV observations from the confirmation paper, which indicates that the orbital period was at least a few years. Following a year of RV monitoring, we were finally able to constrain the orbital period of the outer companion to approximately 2900 days.

### Monitoring TTVs of TOI-201 b

**Fig. 1.**
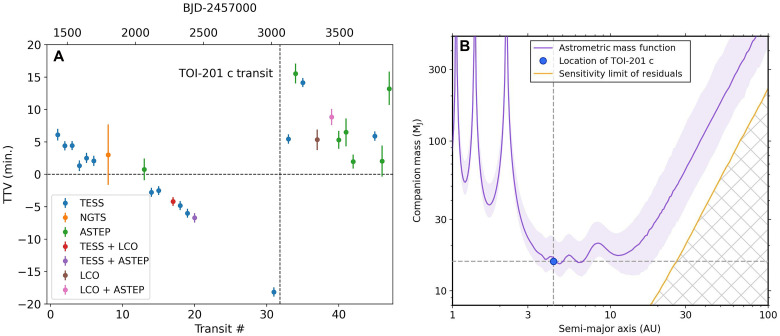
Evidence for TOI-201 c from TTVs and astrometry. (**A**) TTVs for TOI-201 b from TESS and ground-based facilities showing a gradual decline followed by a sudden discontinuity at the time of the outer companion’s transit. (**B**) The astrometric acceleration observed in Hipparcos-Gaia astrometry is consistent with the properties of the ≈15 M_J_ outer companion, and otherwise places limits on more massive companions in the system.

The single transit of the outer companion coincided with the start of significant TTVs of the warm Jupiter. While early transits occur when predicted, the warm Jupiter’s transits immediately before and after the outer companion’s single transit varied, with the one before occurring minutes earlier than predicted and the two after occurring later. These variations could not be explained by a slightly erroneous ephemeris or period, indicating that the warm Jupiter and outer companion are dynamically interacting. Unfortunately, the system would stop being observed by TESS soon after, as it did not observe TOI-201 between Sectors 69 and 86. Accordingly, we monitored the warm Jupiter TTVs using ground-based facilities, namely, the LCOGT and the Antarctic Search for Transiting ExoPlanets (ASTEP) telescope located at Concordia station in Antarctica (*17*, *18*). We observed an additional eight transits using LCOGT and ASTEP and eventually obtained an additional transit from TESS when it re-observed the system. These newest observations showed that the TTVs had decreased from their peak immediately after the single transit and appeared to show a gradual decline as the corrected early transits did, as shown in Fig. 1.

### Modeling the TOI-201 system

We model the host star’s parameters by using archival spectroscopy, photometry, and astrometry to perform an isochrone fit. We find the host star is an F-type star that is slightly larger and hotter than the Sun ($R_{⋆}=1.31\;R_{⊙}$, $M_{⋆}=1.32\;M_{⊙}$, and $T_{⋆}=6423\;K$). We also find that the star is relatively young, although the exact age is not well-constrained at $666_{-442}^{+673}$ Myr.

The host star TOI-201 has been found to be a Hipparcos-Gaia astrometric accelerator, meaning that its proper motion changed noticeably between observations from the Hipparcos and Gaia missions (*19*–*21*). This astrometric acceleration can be attributed to an unseen massive, distant companion. We modeled the plausible range of mass functions across different orbital separations [as in (*19*)], and, as Fig. 1 shows, the properties of TOI-201 c can wholly reproduce the observed acceleration. As such, we also jointly modeled the Hipparcos-Gaia astrometry with the RVs and the single transit from TESS to characterize the orbit of TOI-201 c, allowing us to directly constrain for the outer companion an orbital element inaccessible to the transit and RV methods: the longitude of the ascending node (Ω), the last quantity needed to determine the full three-dimensional orbit of TOI-201 c. We obtained a well-constrained measurement of Ω_*c*_ = 211° ± 11°. Combined with the difference in Ω between TOI-201 b and c constrained by the photodynamical and RV joint fit, we also obtain Ω_*b*_ = 198° ± 10°. To our knowledge, this is the first constraint on the absolute value of Ω for a warm Jupiter planet.

**Fig. 2.**
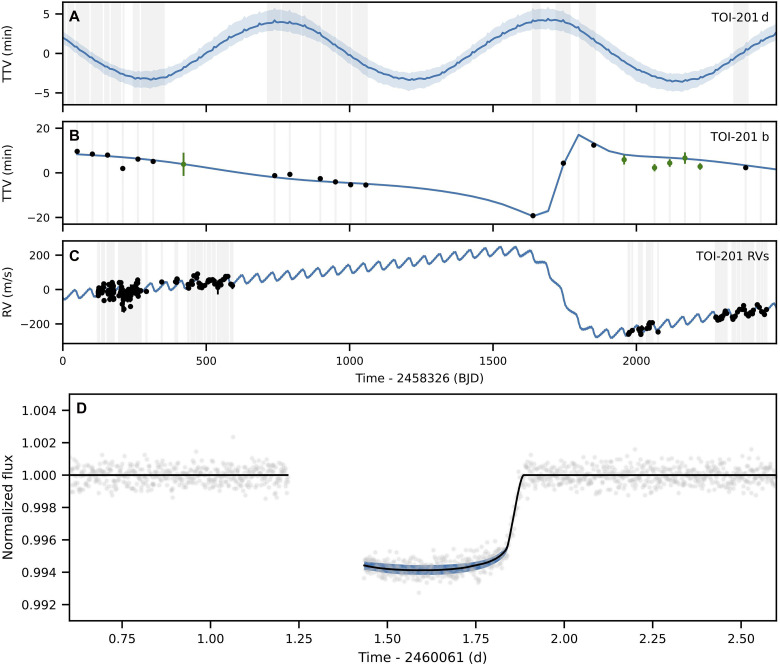
Photodynamical analysis of TTVs and RVs for TOI-201. (**A**) Best-fit TTV model for TOI-201 d. (**B**) TTVs of TOI-201 b from TESS (black points) and ground-based facilities (green points) and best-fit model. (**C**) RV data and best-fit RV model. (**D**) Transit and best-fit model for TOI-201 c. All the panels show the model posterior median as a blue line and the 1-σ posterior uncertainties as light blue shading. In (B) and (C), the uncertainties are smaller than the line width.

We fit the aforementioned TESS photometry, RVs from CORALIE, HARPS, and PFS; LCOGT and ASTEP photometry were acquired for TOI-201 b, alongside with archival LCOGT and Next-Generation Transit Survey (NGTS) photometry for TOI-201 b and archival RVs from FEROS and MINERVA-Australis to determine the orbital and physical parameters of the three known bodies orbiting TOI-201. We used the Python Tool for Transit Variations (pyTTV) to perform photodynamical modeling of the photometry jointly with the RVs. The resulting best-fit parameters and associated uncertainties are listed in Table 1, and the data and best-fit models are shown in Fig. 2.

**Table 1.** TOI-201 stellar and planetary properties. The stellar parameters are derived from our isochrone fit, while the planet parameters are derived from our joint photodynamical-RV fit. The longitude of the ascending node for TOI-201 c comes from our transit-RV-astrometry fit, while the values for b and d come from combining the value for c and the relative longitudes from the photodynamical modeling.

Stellar parameters	Value	Reference
Mass (M_⊙_)	$1.32_{-0.04}^{+0.02}$	This work
Radius (R_⊙_)	1.31 ± 0.01	This work
Luminosity (L_⊙_)	2.61 ± 0.12	This work
Effective temperature (K)	$6423_{+86}^{-90}$	This work
Age (Gyr)	$0.666_{-0.442}^{+0.673}$	This work
**TOI-201 b parameters**		
Orbital period (days)	52.9786 ± 0.0001	This work
Time of inferior conjunction (BJD)	2458376.0521 ± 0.0002	This work
Semi-major axis (AU)	0.303 ± 0.002	This work
Inclination (°)	91.18 ± 0.02	This work
Eccentricity	0.275 ± 0.009	This work
Argument of periastron (°)	83 ± 2	This work
Longitude of ascending node (°)	198 ± 10	This work
Radius (Earth radii)	11.4 ± 0.1	This work
Mass (Earth masses)	164 ± 5	This work
Density (g cm^−3^)	0.61 ± 0.02	This work
**TOI-201 c parameters**		
Orbital period (days)	2890 ± 20	This work
Time of inferior conjunction (BJD)	2460062.59 ± 0.02	This work
Semi-major axis (AU)	4.37 ± 0.04	This work
Inclination (°)	$89.92_{-0.04}^{+0.10}$	This work
Eccentricity	0.651 ± 0.006	This work
Argument of periastron (°)	96 ± 2	This work
Longitude of ascending node (°)	211 ± 11	This work
Radius (Earth radii)	10.4 ± 0.3	This work
Mass (Earth masses)	4990 ± 100	This work
Density (g cm^−3^)	24 ± 2	This work
**TOI-201 d parameters**		
Orbital period (days)	5.84889 ± 0.00009	This work
Time of inferior conjunction (BJD)	2458374.032 ± 0.003	This work
Semi-major axis (AU)	0.0698 ± 0.0005	This work
Inclination (°)	91.7 ± 1.6	This work
Eccentricity	03 ± 0.1	This work
Argument of periastron (deg)	340 ± 80	This work
Longitude of ascending node (°)	175 ± 20	This work
Radius (Earth radii)	1.39 ± 0.07	This work
Mass (Earth masses)	5.8 ± 2	This work
Density (g cm^−3^)	11 ± 4	This work
	
Inclination between b and d (°)	$28.0_{-14.2}^{+10.6}$	This work
Inclination between b and c (°)	$13.4_{-2.3}^{+2.0}$	This work
Inclination between c and d (°)	$41.8_{-16.4}^{+10.9}$	This work

We find very similar values for the orbital parameters (period, eccentricity, and argument of periastron) of the warm Jupiter as the discovery paper, though we obtain tighter constraints on both the eccentricity and argument of periastron. We obtain a mass for the warm Jupiter of 164 ± 5 Earth masses (0.52 ± 0.02 Jupiter masses). For the super-Earth, we obtain a relatively weakly constrained mass of 5.8 ± 2 Earth masses. Combined with its radius of 1.39 ± 0.07 Earth radii, we obtain a relatively high bulk density of 11 ± 4 g cm^−3^, twice that of Earth. We also find that its orbit is moderately eccentric (*e* = 0.3 ± 0.1). The outer companion is the longest-period transiting body found by TESS to date, with an orbital period of 2890 ± 20 days and corresponding semi-major axis of 4.37 ± 0.04 AU. We improve the precision on the period by a factor of 10 compared to the literature (*15*). This will be improved even further with observations of its next transit on 26 March 2031. We determine its mass to be 4990 ± 100 Earth masses, or 15.7 ± 0.3 Jupiter masses, placing it just above the deuterium mass burning limit of ~13 Jupiter masses that separates planets from brown dwarfs (*22*). Its orbit is highly eccentric, with an eccentricity of 0.651 ± 0.006, with its closest and furthest approach from the host star bringing it to closer than Mars’s orbit and further than Jupiter’s, respectively.

We are also able to place constraints on the mutual inclinations between the different planets, which is the three-dimensional angle between the orbital planes of two planets and is given by the equation

$$\text{cos}\Delta i_{1,2}=\text{cos}i_{1}\;\text{cos}i_{2}+\text{sin}i_{1}\;\text{sin}i_{2}\;\text{cos}\left(\Omega_{1}-\Omega_{2}\right)$$

We find that the warm Jupiter and brown dwarf have a mutual inclination of $13.4_{-2.3}^{+2.0}$°, the warm Jupiter and super-Earth have a mutual inclination of $28.0_{-14.2}^{+10.6}$°, and the super-Earth and brown dwarf have a mutual inclination of $41.8_{-16.4}^{+10.9}$°. Our mutual inclination between the warm Jupiter and brown dwarf is notionally over 5σ from zero, and differs by more than 2σ from the value from the literature (*15*). However, when we consider a more limited dataset consisting only of the archival RVs and TESS photometry, we obtain a value consistent with the literature result. Hence, we suggest that the difference from the literature result stems from the larger observational datasets obtained for this work and indicates a moderate but statistically significant mutual inclination between the orbits in the TOI-201 system.

## DISCUSSION

### Evolutionary history

The elevated orbital eccentricity of the brown dwarf is indicative of a dynamically hot past. Several mechanisms are known to increase orbital eccentricity. We rule out interactions with the disk because they require a cavity interior to the brown dwarf’s orbit (*23*), which is not allowed given the existence of the two interior planets. A stellar flyby is highly unlikely due to the unrealistically tight distance of closest approach required to generate the eccentricity of TOI-201 c, and we find that high-eccentricity migration would have resulted in the ejection of TOI-201 d. Two plausible scenarios remain: planet-planet scattering (*24*, *25*) and von-Zeipel-Lidov-Kozai (vZLK) cycles (*26*).

We examined the possibility that the brown dwarf obtained its eccentricity through planet-planet scattering with a now-ejected third giant planet early in the system’s history. We carried out a suite of *N*-body simulations, varying the initial orbital properties of the four bodies as well as the mass of the ejected planet. While it is possible to obtain the observed parameters via this scenario, only ~1% of the simulations reproduce the present-day TOI-201 system. Our methodology and results are described in more detail in Supplementary Text.

The dynamical architecture of the TOI-201 system could be sculpted by an as of yet undetected stellar companion. Such an unseen stellar companion is capable of inciting vZLK oscillations in TOI-201 c (*27*, *28*). Such vZLK cycles can not only explain the high eccentricity of TOI-201 c but also those of TOI-201 b and d, as a result of their interaction with the outer, eccentric giant planet.

We investigated the dynamical influence of a stellar companion on the TOI-201 planets using the *N*-body code REBOUND (*29*, *30*). We selected parameters for our hypothetical stellar companion from the constraints based on analysis of the RVs, high-resolution imaging, and Gaia imaging and astrometry (see fig. S14). Specifically, we adopted a companion mass $M_{\text{comp}}=0.1\;M_{⊙}$, initial orbital semi-major axis sampled uniformly from $a_{\text{comp}}\in \left[80,120\right]$ au, eccentricity $e_{\text{comp}}=0$, and inclination uniformly sampled in $i_{\text{comp}}\in \left[55,65\right]$°. Planet semi-major axes were sampled within their uncertainties, inclinations were sampled between 0° and 5°, the eccentricity for TOI-201 d was sampled uniformly between 0 and 0.1, and eccentricities for TOI-201 b and c were sampled uniformly between 0 and 0.2. The nonzero primordial eccentricities we used can plausibly be excited via interaction with the protoplanetary disk (*31*) and/or through planet-planet dynamical excitation (*32*), which may be a common feature of systems with multiple giant planets such as TOI-201 (*33*).

**Fig. 3.**
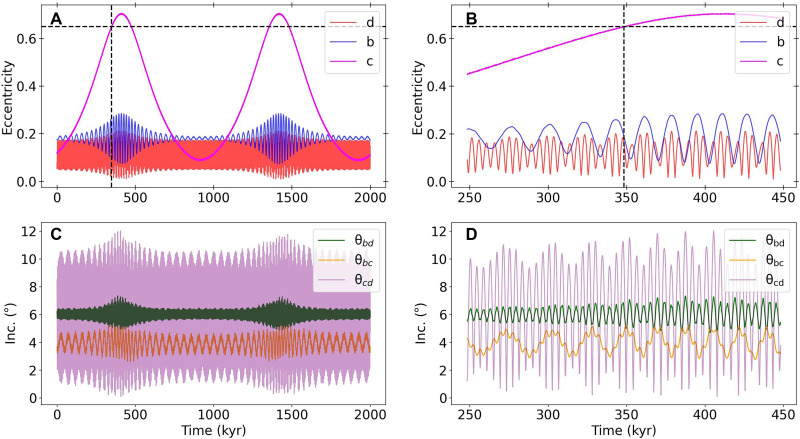
Dynamical evolution of vZLK simulation, which replicates the observed system architectures. The system contains a hypothetical undetected binary companion of mass $0.1\;M_{⊙}$, semi-major axis 102 AU, and initial mutual inclination 64°. Dashed lines in (A) and (B) indicate the first time for which $e_{c}$ = 0.65. (**A**) The evolution of the eccentricities over the span of 2000 kyr (2 Myr). (**B**) Zoom-in of (A) between 250 and 450 kyr when the eccentricity of TOI-201 c first reaches 0.65 showing the short timescale evolution. (**C**) The evolution of the mutual inclinations over the span of 2000 kyr. (**D**) Zoom-in of (C) between 250 and 450 kyr showing the short timescale evolution.

Figure 3 showcases a representative example of the dynamical evolution of the TOI-201 planets in the presence of an outer stellar companion. As expected under vZLK cycles, the eccentricity and inclination of TOI-201 c undergo periodic oscillations over the vZLK timescale ~300 kyr. The observed eccentricity for TOI-201 c, $e_{c}$ ~ 0.65 (see Table 1), is readily attained near the maximum of its vZLK cycle. During its high-eccentricity phases, TOI-201 c pumps the eccentricities and mutual inclinations of the inner planets to their observed values within their respective uncertainties ($e_{b}$ = 0.275 ± 0.009, $e_{d}$ = 0.3 ± 0.1, $\Delta i_{\mathrm{bc}}$ = 13° ± 22°, $\Delta i_{\mathrm{cd}}$ = 41° ± 29°, and $\Delta i_{\mathrm{bd}}$ = 28° ± 28°). Although the resulting mutual inclinations are systematically smaller than the observed values, exploration of a wider range of initial conditions (e.g., an initially misaligned inner system or greater companion inclination) could reveal more extreme misalignment.

The vast majority of our simulated systems remain dynamically stable over Myr timescales, the longest duration of integration we explored. We conclude that vZLK oscillations are the most plausible explanation for TOI-201 c’s high eccentricity.

Follow-up observational work could verify the vZLK hypothesis if a stellar companion is found. Measurements of TOI-201’s stellar obliquity could also validate the vZLK scenario, since we would expect the orbital angular momentum vector of TOI-201 c to currently be misaligned with the host star’s spin axis.

Last, it is possible that a combination of planet-planet scattering and vZLK oscillations could also explain TOI-201’s architecture, a possibility which should be explored in future studies of this system.

### Current state and immediate future of the system

Our goal in this section is to characterize the stability and secular dynamical evolution of the planetary system. We show that while the system is likely stable, there is a non-negligible chance of planet d experiencing instability over Myr timescales. We highlight that TOI-201 exhibits significant secular dynamical evolution over human-observable timescales (~decades); long-term observation of TOI-201 may therefore provide an unprecedented glimpse into the active lives of planetary systems in real time.

To explore the current state and future evolution of the TOI-201 system, we constructed a suite of 500 REBOUND *N*-body calculations (*29*). Planet orbital elements were sampled within their uncertainties from the posteriors, and orbital integrations were performed for up to 2 Myr.

We used the MEGNO chaos indicator from REBOUNDx (*34*) to determine if the system is likely unstable in the present. We find MEGNO scores consistent with stability (see fig. S10). System stability is also reflected in our *N*-body simulations, for which only 1% experienced an instability leading to tidal disruption/ejection of planet d during the high-eccentricity vZLK epochs. There is thus a small but nonzero chance of planet d experiencing dynamical upheaval/destruction over Myr timescales.

**Fig. 4.**
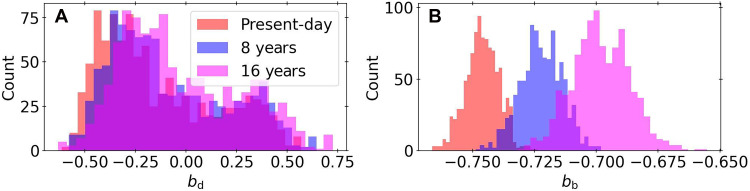
Short-term evolution of the impact parameters of the two inner planets for 1000 integrations of the posteriors. (**A**) The impact parameter of TOI-201 d at the present-day and 8 and 16 years later. (**B**) The same as (A) but for TOI-201 b.

As illustrated in Fig. 4, the transit impact parameters and corresponding transit duration variations of planets b and d evolve significantly over ~decades. This can be confirmed observationally after the next periastron passage of the brown dwarf in 2031, when TOI-201 b’s impact parameter will increase by more than 3-σ from its currently measured value. While planet d’s impact parameter is also evolving rapidly, its shallow transits result in a large uncertainty for its impact parameter, and thus it will be over 200 years before it deviates by 3-σ from its present value. The significant secular evolution of the planetary transits is driven by the planets’ large mutual inclinations. We find that planets b, c, and d will cease to cotransit after just ~200 years, and will only re-establish cotransiting geometry after ~10 kyr (fig. S9). Changes in transit geometry are particularly acute for planet b, which exhibits step-like perturbations excited at each periapse passage of TOI-201 c. Our integrations therefore indicate that continued monitoring of the system could witness the evolution of the warm Jupiter’s stellar obliquity as it is sculpted by the outer brown dwarf. Follow-up measurements of the stellar obliquity are called for. This can be achieved through observations of the Rossiter-McLaughling (RM) effect; for TOI-201 b, the expected RM effect amplitude is ~30 m/s, which is well within the capabilities of current spectrographs.

The TOI-201 system further underscores how three-dimensional orbital characterization can shed light on the active lives of planetary systems; without three-dimensional orbit information, we find that the system does not evolve sufficiently quickly that we are be able to watch its architecture undergo dynamical sculpting in real time.

## MATERIALS AND METHODS

### TESS photometry

In addition to the 14 initial sectors used in the TOI-201 b discovery paper (*1*), we use the most recent 18 sectors of TESS photometry obtained for TOI-201 (TIC 350618622). These 18 sectors include nine new transits for the previously identified warm Jupiter TOI-201 b, for a total of 16 transits observed by TESS. The TOI-201 data observed at 2-min cadence and the image data were reduced and analyzed by the Science Processing Operations Center (SPOC) (*35*) at NASA Ames Research Center. The TESS Science Office reviewed the vetting information and issued an alert on 7 May 2019 for TOI-201 b and on 11 March 2020 for TOI-201.02 (*13*). The signals have been repeatedly recovered with different observations. Combining multiple sectors, the SPOC conducted a transit search of Sectors 1 to 68 on 30 October 2023 with an adaptive, noise-compensating matched filter (*36*–*38*), producing Threshold Crossing Events for which an initial limb-darkened transit model was fitted (*39*) and a suite of diagnostic tests were conducted to help make or break the planetary nature of the signals (*40*). The host star is located within 0.62 ± 2.49 arc sec of the source of the transit signal for TOI-201 b and within 4.29 ± 4.56 arc sec for TOI-202.02. The transit signatures were also detected in searches of Full Frame Image data by the Quick-Look Pipeline (QLP) at MIT (*41*, *42*). This candidate was not confirmed alongside the warm Jupiter as a one-planet RV model incorporating Gaussian processes for stellar variability was preferred over a two-planet model (*1*). However, we find that the signal attributed to stellar variability is better explained by a long-period planet. We visually identified a single, partial transit-like dip in Sector 64 unrelated to TOI-201 b. We use the presearch data conditioned simple aperture photometry (PDCSAP) (*43*–*45*) light curve for the modeling of the majority of the TESS data. Most of a transit of planet b that occurred in Sector 8 occurred while the instrument was turned off. The transit egress occurred as observations resumed when temperatures were still changing, resulting in a large ramp feature present in both the simple aperture photometry (SAP) (*46*, *47*) and PDCSAP light curves (see fig. S1). There is also a downlink gap at the start of the single transit in Sector 64 meaning the ingress was not observed. The PDCSAP flux light curve exhibits a steep slope that can be mistaken for an ingress. This feature is not present in the SAP light curve or the QLP (*41*, *42*) light curve, indicating that it is an artifact of the PDC process. As such, we use light curves corrected using cotrending basis vectors (CBVs) as described next, to model the partial transit of TOI-201 b in Sector 8 and the single transit of TOI-201 c in Sector 64 (fig. S1). We used software from (*48*) that performs systematic corrections and automatically optimizes parameters for correctors available in this code. Here, we focus on a corrector that is a version of PDC adapted within the CBVCorrector class of Lightkurve (*49*). This corrector uses the CBV technique that the PDC method of the SPOC pipeline uses. Hereafter, we will refer to this corrector as CBV (for comparison purposes, results from other correctors in the code are also shown in fig. S1; for further details on these correctors, see the code references above). Flux fraction and crowding adjustments are applied to the corrected light curves. To automatically select optimal values for a set of parameters of the CBVCorrector, each corrected light curve is evaluated using the Savitzky-Golay combined differential photometric precision (sgCDPP) proxy algorithm discussed in (*50*, *51*) and implemented in Lightkurve, for various durations (see the top panels in fig. S1). For a grid of corrector parameter values, the code calculates the harmonic mean (HM) of these sgCDPPs of various durations and selects the corrected light curve that minimizes the HM.

As the single transit in Sector 64 was not associated with either the super-Earth candidate or the confirmed warm Jupiter, the orbital period of the outer candidate was almost completely unconstrained. However, the vast amount of TESS data meant we could determine the minimum orbital period as well as test narrow windows associated with periods where potential additional transits could have fallen into data gaps. To do this, we used the MonoTools package (*52*, *53*) to determine which orbital periods were permitted by the TESS data (see fig. S2). The package determines the periods allowed by the photometry and calculates a probability for each based on the geometric transit probability and a prior on the eccentricity needed to match the transit duration based on the eccentricity distribution of known planets. For systems with multiple transiting planets, MonoTools automatically used an eccentricity prior derived from transiting Kepler planets (*54*); however, this is only valid for compact systems of small planets. This typically results in the posterior period distribution being more tightly distributed around the circular period estimated from the transit model.

### Ground-based photometry

We observed potential TTVs in the transits observed by TESS in Sectors 61, 65, and 68 around the time of the single transit event. To determine whether the TTVs were real and characterize them if they were, we observed 15 transits of TOI-201 b from the ground using a combination of the NGTS survey 0.2-m telescopes (*55*) located at ESO’s Paranal Observatory, the LCOGT (*56*) 1.0-m network nodes at Cerro Tololo Inter-American Observatory in Chile (CTIO), Siding Spring Observatory (SSO) near Coonabarabran, Australia, and South Africa Astronomical Observatory (SAAO) near Sutherland, South Africa, and the ASTEP telescope located at Concordia station in Antarctica (*57*, *58*). The LCOGT images were calibrated by the standard LCOGT BANZAI pipeline (*59*) and differential photometric data were extracted using AstroImageJ (*60*).

We observed two transit windows of TOI-201 d using LCOGT-CTIO and LCOGT-SAAO. The transit event is generally too shallow to be detected by ground-based telescopes. However, we ruled out nearby eclipsing binaries as potential sources of the detection in the TESS data.

We also attempted to search for additional transit events of the brown dwarf TOI-201 c, before its period was known. We searched the shortest possible periods as determined by our MonoTools analysis. We collected nearly two dozen observations over the time period 20 November 2023 to 20 December 2024 using LCOGT-CTIO, LCOGT-SSO, LCOGT-SAAO, the PEST located near Perth, Australia, Hazelwood Observatory near Churchill, Victoria, Australia, HATPI located at Las Campanas Observatory in the Chilean Andes, and from three Unistellar Network telescopes in Australia and New Zealand (*61*). We found no transit-like events in the periods we checked, which were different from the period determined in this work. An observation log of all ground-based lightcurve observations is provided in Table 2.

**Table 2.** Ground-based light curve observations of TOI-201. The transits of TOI-201 c were not detected as the observations were made before the true orbital period was known and these tested shorter orbital periods. The transits of TOI-201 d were not detected as they are too shallow for the instruments used, although we could rule out nearby eclipsing binaries (NEBs) as the source of the events.

Telescope	Planet	Date	Filter(s)	Comments
NGTS	b	19 September 2019	NGTS^*^	Ingress
LCOGT-CTIO	b	9 January 2021	Y^†^	Full transit
LCOGT-SSO	b	4 December 2023	Y	Ingress
LCOGT-SAAO	b	4 December 2023	Y	Egress
LCOGT-SAAO	b	19 March 2024	Y	Egress
LCOGT-SSO	b	1 February 2025	Y	Out-of-transit
ASTEP	b	11 June 2020	None	Egress
ASTEP	b	17 June 2021	None	Egress
ASTEP	b	28 June 2023	~BP^‡^, ~RP^§^	Full transit
ASTEP	b	19 March 2024	~BP, ~RP	Ingress
ASTEP	b	11 May 2024	~BP, ~RP	Full transit
ASTEP	b	3 July 2024	~BP, ~RP	Full transit
ASTEP	b	25 August 2024	~BP, ~RP	Full transit
ASTEP	b	25 March 2025	~BP, ~RP	Egress
ASTEP	b	17 May 2025	~BP, ~RP	Full transit
LCOGT, PEST, HATPI, Unistellar Network, Hazelwood Observatory	c	29 November 2023 to 20 December 2024	Various	Various periods ruled out (see text)
LCOGT-CTIO	d	4 November 2020	zs^¶^	No NEBs
LCOGT-SAAO	d	1 January 2021	zs	No NEBs

* Custom filter with bandpass 5200 to 8900 Å.

† [Table-fn T2F2]Pan-STARRS Y-band (λ_c_ = 10,040 Å, width = 1120 Å).

‡ [Table-fn T2F3]Similar to Gaia – BP band.

§ Similar to Gaia – BP band.

¶ Pan-STARRS *z_s_* band (λ_c_ = 8700 Å, width = 1040 Å).

### Spectroscopic observations

We use archival RV measurements from CORALIE, HARPS, FEROS, and MINERVA-Australis in combination with new observations from CORALIE, HARPS, and PFS to characterize the system.

### CORALIE and HARPS

We collected 23 new RV measurements between UT 02 January 2024 and UT 13 April 2025 with the CORALIE spectrograph on the Swiss 1.2-m Leonhard Euler Telescope at the ESO La Silla Observatory in Chile (*62*). We also obtained 14 new measurements between UT 20 October 2024 and UT 30 March 2025 with the HARPS spectrograph on the ESO 3.6-m telescope, also at La Silla Observatory (*63*). We also include the 13 and 42 archival RVs used in the TOI-201 b discovery paper from CORALIE and HARPS, respectively.

### Planet Finder Spectrograph

We collected 19 RV measurements of TOI-201 between UT 20 December 2023 and UT 03 March 2024 with the Carnegie PFS (*64*–*66*). PFS is a high-precision echelle spectrograph attached to the 6.5-m Magellan Clay telescope at Las Campanas Observatory in Chile. It has a spectral resolution of 130,000 and covers the 390- to 734-nm spectral window. Wavelength calibration is carried out using an iodine absorption cell, which also allows for characterization of the instrumental profile. Spectra were reduced using the standard PFS reduction pipeline (*64*, *67*) and RV measurements were extracted using a custom IDL pipeline.

### Archival FEROS and MINERVA-Australis RVs

Our analysis also includes 52 archival RVs from the Fiber-fed Extended Range Optical Spectrograph (FEROS) at the MPG/ESO 2.2-m telescope at La Silla Observatory (*68*) and 62 of those from the MINERVA-Australis telescope facility at Mount Kent Observatory in Queensland, Australia (*69*).

### Astrometry

TOI-201 has been observed by the astrometric space missions Hipparcos and Gaia, active between 1989 to 1993 and 2014 to 2025, respectively. This allows us to use cross-calibrated proper motion data from Hipparcos-Gaia astrometry (*19*, *20*) to constrain the reflex motion caused by TOI-201 c over a ~25-year baseline.

We extracted the proper motion data for TOI-201 from the Gaia EDR3 version of the Hipparcos-Gaia Catalog of Accelerations (*21*). In this catalog, the default linear proper motion hypothesis has a $\chi^{2}$ goodness-of-fit statistic of 40, which is one of the highest values for any confirmed TESS planetary system. In physical units, this is equivalent to a net change in tangential velocity of 134 ± 15 m s^−1^ between the Gaia proper motion and the mean proper motion in the interval between Hipparcos and Gaia observations. As shown in Fig. 1, the observed astrometric acceleration is consistent with the signal expected from TOI-201 c.

### Stellar characterization

We use the effective temperature, surface gravity, and metallicity from the TESS Input Catalog along with the Gaia DR3 parallax and magnitudes (G, BP, and RP), Two Micron All Sky Survey (2MASS) magnitudes (J, H, and KS), and Wide-field Infrared Survey Explorer (WISE) magnitudes (W1, W2, and W3) to perform an isochrone fit to constrain further the spectroscopic parameters and derive the physical parameters of the host star. The spectroscopic parameters, parallax, and magnitudes are used as priors to determine the goodness of fit. We use the isochrone package (*70*) to generate the isochrone models used to sample the stellar parameters and find the best-fit parameters by using a Markov Chain Monte Carlo (MCMC) routine using the emcee package (*71*). The routine consists of 40 independent walkers each taking 5 × 10^4^ steps, of which the first 2000 are discarded as burn-in. We find that the host star is a relatively young F star, with an age of $666_{-442}^{+673}$ Myr. The fitted spectroscopic parameters and derived physical parameters, including stellar age, of the host star are reported in table S1.

We use a Generalized Lomb Scargle periodogram (*72*) to search for periodic stellar variability signals in the TESS light curve after masking out all transits. We detect no consistent period in the TESS photometry, with statistically significant periodicities ranging from less than 1 to more than 10 days depending on the sector analyzed.

### Statistical validation of the super-Earth

We rule out false-positive scenarios and statistically validate the super-Earth using the triceratops package (*14*, *73*), including the contrast curve from archival SOAR high-resolution imaging to provide additional constraints on the stellar companions generated. We calculate a false-positive probability and nearby false-positive probability of 0.008 and 1 × 10^−12^, respectively. Given these values, TOI-201 d is a statistically validated planet.

### Preliminary RV model

We initially modeled the radial velocities using the radvel package (*74*). We obtained a preliminary orbital solution for the parameters of the outer companion and a tentative mass measurement for the inner super-Earth. We obtained a somewhat well-constrained period, eccentricity, and mass for the brown dwarf that were consistent with both the modeling incorporating the transits and astrometry and the final full photodynamical modeling. We also obtained a mass of 11 ± 4 $M_{⊕}$ for the super-Earth, which yielded a physically improbable density of 22 g cm^−3^, or four times Earth’s density.

### Joint model incorporating astrometry

With the overall system architecture of the TOI-201 system having been determined from RV and transit data, we next perform a joint model incorporating the Hipparcos-Gaia astrometry. This model is based on the one developed in (*75*) to jointly model RVs and Hipparcos-Gaia astrometry, implementing modifications for handling multiplanet systems from (*76*). To our knowledge, TOI-201 c is the first substellar companion to be detected simultaneously in RV, transit, and astrometry data. To incorporate the transit data in this model, we use the batman package (*77*) to generate transit models.

Since this model assumes Keplerian dynamics, we cannot straightforwardly account for TTVs arising from inter-planet interactions. These effects have no impact on the astrometry at the level of precision; so for the sake of simplicity, we restrict the included photometric data to a single transit each for TOI-201 b and TOI-201 c. We also choose to omit TOI-201 d from this model since its contribution to RV variability is small and to the astrometry negligible. We assume that TOI-201 b does not significantly contribute to the astrometry, which is reasonable as its orbital period is significantly shorter than the 3-year observing baselines of both Hipparcos and Gaia DR3.

This model includes a total of 32 variable parameters, of which seven describe the star and system (stellar mass $M_{*}$, stellar density $\rho_{*}$, quadratic limb-darkening coefficients $u_{1}$, $u_{2}$, parallax ϖ, and barycentric proper motions $\mu_{\alpha ,\text{bary}}$ and $\mu_{\delta ,\text{bary}}$), 10 describe the zero-point offsets and jitter terms for the five RV datasets, and the remaining 15 describe the properties of TOI-201 b and TOI-201 c. These parameters are the orbital period *P*, the mass $M_{p}$, the eccentricity and argument of periastron parameterized as $\sqrt{e}\text{sin}\omega$, $\sqrt{e}\text{cos}\omega$, transit time $T_{0}$, impact parameter *b*, and radius ratio $R_{p}/R_{*}$. For TOI-201 c, we have additionally the longitude of node Ω, which is used exclusively for fitting to the astrometry.

We reproduce the posterior parameters from this model in table S2 and in fig. S3 the corresponding fit to the RVs, Hipparcos-Gaia astrometry, and the transit of TOI-201 c. It can be seen in the Hipparcos-Gaia astrometry that the high significance of proper motion the nonlinearity reported in the Hipparcos-Gaia Catalog of Accelerations (*21*) arises in large part from the coincidence of the Gaia observations with the previous periastron passage of TOI-201 c, which occurred at BJD 2457241 ± 47 [~August 2015; compare (*78*)]. As a result, the astrometry helps to provide a robust constraint on the orbital period of the outer companion. For TOI-201 c, we find key parameters of $P=2834_{-38}^{+43}$ d, $e=0.610_{-0.041}^{+0.047}$, ω = 98.0° ± 6.2°, and $M_{p}=15.4_{-0.8}^{+1.0}\;M_{J}$. The bulk of the posterior constraint on the orbital inclination comes from the transit, rather than the astrometry ($i=89.916_{-0.022}^{+0.044}$°); this means that the main degree of freedom constrained by the astrometry is the longitude of node, which we uniquely determine to be Ω = 212° ± 11°.

Beyond the detection of TOI-201 c, the ~25-year-long temporal baseline of the Hipparcos-Gaia astrometry allows us to place limits on the presence of other massive companions in the system. Subtracting our best-fit proper motion model, the 3-σ upper limit on the remaining Gaia tangential velocity anomaly is <45 m s^−1^. We show the mass detection limits from this constraint in Fig. 1, where companions above the orange line are notionally excluded. In reality, the residual of the astrometric model for TOI-201 c is liable to overconstraint due to the limited scope of the astrometric data, so this detection limit is likely to be optimistic; nonetheless, we believe it is reasonable to infer from the astrometric constraints that stellar-mass companions (≳80 M_J_) to TOI-201 can be largely ruled out for projected separations within ≲50 AU.

### Photodynamical analysis of photometry and RVs

Since TOI-201 b shows strong TTVs induced by the periastron passage of an eccentric outer companion, similar to Kepler-419 b (*79*, *80*), Kepler-448 b, and Kepler-693 b (*81*), we performed a joint photodynamical analysis of the TESS photometry, the ground-based photometry, and the RVs. The analysis was done using the PyTTV following the methodology described in (*82*) and (*83*), assuming a three-planet configuration. We modeled the light curves corrected using the CBV corrector (described above) for Sectors 8 and 64, the TESS SAP light curves with the 2-min cadence for Sectors 1 to 7, 10 to 13, and the 20-s cadence for the remaining sectors. The ASTEP ground-based photometry has been binned to 1.5 min. The model is parametrized as described in (*82*). The model is parameterized using the sampling parameters $\sqrt{e}\text{cos}\omega$ and $\sqrt{e}\text{sin}\omega$ with half-normal priors on the orbital eccentricities. Since the radvel analysis gives an unrealistically high mass for the innermost planet, we carried out two photodynamical analyses assuming different priors on the planet mass. In the first case, we set a uniform prior on the log_10_ planet mass from −5 to −4.1, and in the second case, we set a normal prior on the 1og_10_ planet mass with a mean of −5.07 and SD of 0.15. The latter one was calculated using the spright mass-radius relation from (*84*). We considered two scenarios for the prior set on the longitude of the ascending nodes. First, we carried out an analysis fixing $\Omega_{d}$ to zero and setting uniform priors on $\Omega_{b}$ and $\Omega_{c}$. Second, we carried out an analysis with a normal prior, N(212,11), on $\Omega_{c}$, as determined from the astrometry in the preceding analysis, and the longitudes of the ascending nodes of both inner planets were set free. In addition to the main photodynamical analysis, we tested for mass-eccentricity degeneracies by carrying out several photodynamical analysis scenarios assuming different priors on planet masses and eccentricities following (*85*). In particular, we used their default prior (log-uniform in planet masses and uniform in eccentricities) and their high-mass prior (uniform in planet masses and log-uniform in eccentricities), which pull the solution toward opposite ends of the degeneracy (*86*). The model parameters and their priors for the main analysis are listed in table S3.

The PyTTV photodynamical code simultaneously models photometric and RV data using REBOUND (*29*, *30*, *87*) for dynamical integration. It incorporates general relativity effects through REBOUNDx (*34*) and accounts for the light travel time effect (*88*). Transit modeling is performed using PyTransit (*89*–*91*). The analysis begins with global optimization using the differential evolution algorithm (*92*, *93*). This optimization is followed by MCMC sampling, starting from the global optimization results using the emcee sampler (*71*). Correlated photometric noise is modeled as a Gaussian process, implemented using the Celerite package (*94*).

**Fig. 5.**
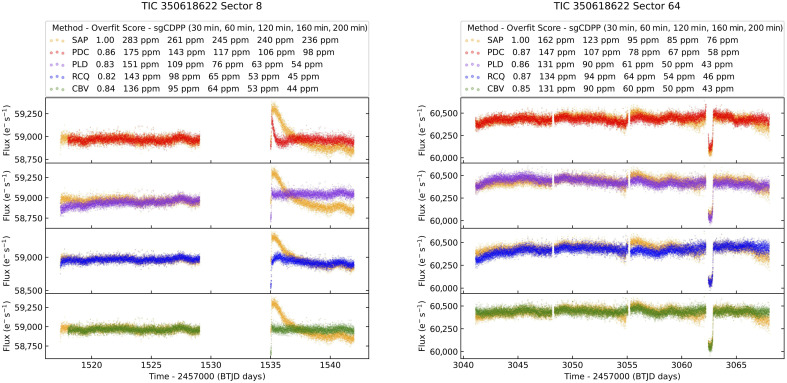
Data and individual transits of TOI-201 b in the photodynamical model. The transits are shown in chronological order.

We show the modeled TTVs for TOI-201 d and TOI-201 b and the modeled RVs for the system in Fig. 2. In the second panel, we also show the measured TTVs for TOI-201 b, as well as the RV measurements in the lowest panel. The individual transits are shown in Fig. 5 for TOI-201 b. The phase-folded plot for TOI-201 d is shown in fig. S4. The photodynamical analysis with the uniform prior on the innermost planet’s mass leads to a mass estimate that agrees with the values from the radvel analysis. Since this mass value corresponds to an unrealistically high planet density, we report the solution using the normal prior on the planet mass as the final solution in table S4. The two Ω scenarios yielded identical posteriors for all the parameters except for the three Ωs. We note that our posterior solution shows a degeneracy in impact parameter for TOI-201 c and its transit duration (fig. S5). This also creates a degeneracy in the impact parameter and transit center time. Observation of a full transit of TOI-201 c is required to solve these degeneracies. The next transit opportunity is 26 March 2031 at 07 UT with an uncertainty of 21 hours. The longitudes of the ascending nodes of both TOI-201 b and TOI-201 c are nearly but not exactly aligned, leading to a mutual inclination of $I_{\text{bc}}$ = 13° ± 2°. There is no significant evidence for nonzero mutual inclinations between the innermost planet and the two outer companions. The limb-darkening parameter, $q_{2}$, is not well constrained, and therefore we report only the 95th percentile of its posterior distribution.

## Supplementary Materials

### The PDF file includes:

Supplementary Text Figs. S1 to S15 Tables S1 to S7 Legends for data S1 to S5 References
