# Supplementary material for: Uncovering the rapidly evolving orbits of the dynamic TOI-201 system
Source: Sci Adv. 2026 Apr 15;12(16):eaef2618. doi: 10.1126/sciadv.aef2618 (PMC13082327; doi:10.1126/sciadv.aef2618)
Supplement: Supplementary file 1 — Supplementary Text Figs. S1 to S15 Tables S1 to S7 Legends for data S1 to S5 References [file sciadv.aef2618_sm.pdf]

Science Advances
AAAS

Supplementary Materials for

Uncovering the rapidly evolving orbits of the dynamic TOI-201 system

Ismael Mireles *et al.*

Corresponding author: Ismael Mireles, mirelesi@unm.edu

Sci. Adv. **12**, eaef2618 (2026)
DOI: 10.1126/sciadv.aef2618

The PDF file includes:

Supplementary Text
Figs. S1 to S15
Tables S1 to S7
Legends for data S1 to S5
References

Other Supplementary Material for this manuscript includes the following:

Data S1 to S5

Supplementary Text

Present-day dynamics

In this section our goal is to characterize the present-day dynamics of the TOI-201 system. Through a suite of numerical orbit integrations, we show that TOI-201 is a relatively stable system that nonetheless possesses a non-zero chance of chaotic tidal destruction of planet d. We also demonstrate that the TOI-201 system exhibits secular dynamical changes in planetary transits over timescales directly observable by humans.

To study the present-day dynamics of the TOI-201 system, we sample the system's orbital parameters from the derived posterior distributions summarized in Table S4 and integrate using the N-body code `REBOUND` (*29*). Specifically, we use the integrator `WHFast` (*30, 96*) and choose a timestep which is 1/50th of the innermost planet's orbital period to ensure numerical stability. We conduct two sets of integrations: Sample A consists of 500 simulations, each spanning 1 Myr, while Sample B includes 100 simulations, each for 10 Myr. The sampling of the posterior distributions is independent between Samples A and B.

We find in both Samples A and B that the high eccentricities and mutual inclination of TOI-201 b and c result in high-amplitude secular oscillations in the inclination and eccentricity of TOI-201 b and d's orbits (Figures S6, S7). These secular interactions result in the excitation of TOI-201 d onto a moderately eccentric ($e \sim 0.2 - 0.5$) orbit. In Sample A, the mean amplitude of eccentricity oscillations of TOI-201 b and TOI-201 d (defined as $e_{\max} - e_{\min}$) is 0.13 and 0.31, respectively. In $\sim 5\%$ of integrations in Sample A, secular interactions result in immediate chaotic evolution of TOI-201 d's eccentricity and subsequent collision with the host star. There are no clear constraints on the posterior distribution if we eliminate the unstable initial conditions. We hypothesize that these systems are in close proximity to secular resonances and that secular analysis may be used to further constrain the posterior distributions (*97*).

Interestingly, a small proportion of systems (~ 3 %) from Sample B which are stable on the 100 kyr timescale exhibit chaotic growth of TOI-201 d's eccentricity after 500 kyr. To determine whether this chaotic behavior may lead to the formation of an ultra-short period planet in a similar scenario to that explored in (*98*), we carry out additional integrations that account for equilibrium tides and general relativity and result in suppression of high ($e \gtrsim 0.9$) eccentricities. Specifically,

we use the `gr_potential` and `tides_constant_time_lag` (*99*) modules in `REBOUNDx` (*34*) and choose TOI-201 d to have tidal Love number $k_2 = 0.4$ and quality factor $Q \sim 10^4$, consistent with a sub-Neptune composition (*100*). We find that these effects are not enough to quench the eccentricity excitations driven by secular chaos, and thus TOI-201 d may (in rare circumstances) undergo tidal disruption on the $\sim$ Myr timescale, unless it is spared by more extreme tidal effects such as chaotic dynamical tides (*10, 101*).

Given the strong eccentricity oscillations of TOI-201 b and c, it is reasonable to question whether either or both bodies may be undergoing tidal migration. Assuming $Q \sim 10^5$ and $k_2 = 0.25$ for both TOI-201 b and TOI-201 c, we find that neither body is currently experiencing tidal migration on astrophysically relevant timescales via equation 3 of (*102*), assuming upper bounds on both orbital eccentricities of 0.7 (as motivated by our earlier integration results). We also investigated whether TOI-201 b and c are undergoing tidal migration due to an additional, undetected planet in the system contributing to dynamical excitation. To determine whether the system could host an additional planet between the orbits of TOI-201 b and c, we use a Mean Exponential Growth of Nearby Orbits (MEGNO) map implemented in `REBOUND` (*103*). Specifically, systems whose MEGNO score is $\gtrsim 3$ for a 10 kyr integration are chaotic and inferred to be unstable on astronomical timescales. With this criterion, we find no stable orbits of super-Earth, Saturn, or Jupiter-sized objects between TOI-201 b and c.

We also find that strong secular and quasi-secular interactions induce meaningful changes in the inclinations of TOI-201 b and d on human-observable ($\sim$ 10 yr) timescales. Specifically, we study the short-term evolution of the transit impact parameter $b = a \cos i / R_\star$ for each planet. Each planet is transiting only when its impact parameter satisfies $|b| < 1$. We find that secular interactions between TOI-201 b, c and d result in substantial changes to the impact parameters of TOI-201 b and d, denoted b_b and b_d, over decade timescales (see Figure S8). Furthermore, there are periodic impulses in b_b which correspond to the periastron of TOI-201 c. These quasi-secular perturbations are, in principle, detectable in as soon as 8 years (Figure 4).

Among the integrations in Sample A, the mean times for TOI-201 d and TOI-201 b to cease transiting are 275 and 615 yr, respectively. Furthermore, the mean time for the system to return to a cotransiting configuration after one planet's impact parameter exceeds unity is 21 kyr. The dynamics of each of the planet's impact parameters is shown in Figure S9.

Evolution of the TOI-201 system

Several aspects of the TOI-201 system make its dynamical history difficult to explain given conventional channels of planet formation. Disk interactions typically only excite eccentricities as high as $e \sim 0.4$ (*104, 105*), unless the inner edge of the disk is truncated (*23*); however, this scenario is in tension with the presence of TOI-201 b and TOI-201 d which are on tighter orbits than TOI-201 c. Here, we evaluate several mechanisms which could explain the architecture of the TOI-201 system, including high-eccentricity migration, stellar fly-bys, planet-planet scattering, and von-Zeipel-Lidov-Kozai (vZLK) cycles.

We find that the existence of TOI-201 d on a tight orbit rules out the possibility of high eccentricity tidal migration (e.g. (*9, 106, 107*)) as a formation channel of TOI-201 b. In particular, we use a MEGNO map in `REBOUND` and sample regions of parameter space with hypothetical proto-TOI-201 b's on orbits consistent with the planet's observed angular momentum but higher eccentricity. We find that for $e \gtrsim 0.8$, TOI-201 d is ejected on the $\sim$ 100 year timescale. A similar analysis, combined with previous established bounds on the tidal capture of brown dwarfs (*108, 109*) rules out the possibility of TOI-201 c having undergone high-eccentricity tidal migration or tidal capture in its dynamical history.

Through a suite of N-body simulations, we find that pumping the eccentricities of TOI-201 b and c through a stellar flyby requires a very tight distance of closest approach of $\sim 4-6$ au (Figure S11). To establish whether such flyby distances are feasible in typical cluster environments, we estimate the timescale to achieve such a flyby following $t_{\rm flyby} \sim (n_\star \sigma_\star d_{\rm flyby}^2)^{-1}$ where $n_\star$ is the stellar density, $\sigma_\star$ is the velocity dispersion, and $d_{\rm flyby} \sim 5$ au is the flyby distance. In young open clusters, $n_\star \sim 10$ pc^{-3} and $\sigma_\star \sim 3$ km s^{-1} (*110*), which implies that $t_{\rm flyby} \gg$ Gyr. We therefore conclude that flybys sufficiently close to excite the eccentricities of TOI-201 b and c are unlikely to occur.

Having shown that flybys are unlikely to excite the eccentricities of TOI-201 b and c, we next consider the possibility of planet-planet scattering as a mechanism for generating the high eccentricities of TOI-201 b and c. Planet-planet scattering is hypothesized to be an important pathway for the sculpting of planetary systems, due to the observed diversity of eccentricities of giant planets and the inferred compactness of configurations formed via core accretion (*8, 24*).

To evaluate the plausibility of the planet-planet scattering scenario, we carry out a suite of

N-body simulations. Specifically, we initialize TOI-201 b and introduce a hypothetical TOI-201 e with mass 7 M_{jup} and semi-major axis sampled uniformly from 4 to 4.5 au. TOI-201 c's orbit is initialized such that it has a mutual Hill spacing $\Delta = 3$ with the hypothetical TOI-201e. We neglect considerations of TOI-201 d to improve simulation runtime. All orbits are initially approximately coplanar and moderately eccentric, with mutual inclinations $\lesssim 3$ deg and eccentricities between 0 and 0.2. We use the hybrid integrator `mercurius` (*111*) to ensure numerical stability despite close encounters, and carry out 4000 integrations of the initial conditions for 100 kyr each.

In 30% of the simulations, the hypothetical TOI-201 e is ejected and the eccentricities of TOI-201 b and c are excited. Among these simulations, $\sim$ 0.5% attained a final eccentricity of TOI-201 c of at least 0.6 (Figure S13). One such example is depicted in Figure S12. Notably, we find that the timescale for which the hypothetical TOI-201 e is ejected is typically 1 – 10 kyr, which is several orders of magnitude less than the inferred age of the system. Therefore, due to the rapid instability timescale and relative paucity of high-eccentricity TOI-201 c's in our simulations, we find that planet-planet scattering may be plausible but requires a narrow range of initial conditions to replicate the system's observed architecture. Future studies could better evaluate the feasibility of this scenario by testing a wider range of initial conditions, which may lead to longer instability timescales or a greater efficiency of exciting TOI-201 c's eccentricity.

Possible Stellar Companions

If the TOI-201 system is undergoing vZLK cycles due to a second star in the system, then this additional stellar companion would have been missed by existing observations. In order to quantify any possible undetected stellar companions, we use Multi-Observational Limits on Unseen Stellar Companions (MOLUSC) to generate a sample of potential companions consistent with the combination of the high-resolution imaging, RV data, Gaia astrometry (in the form of the RUWE), and Gaia imaging (*112*). Of the 100,000 objects we generated, 20% are consistent with the existing observations. The vast majority of these objects have masses less than 0.8 $M_{\odot}$, and most objects more massive than that have semi-major axes that would disrupt the orbits of the planets (see Figure S14). For the remaining stellar mass objects, the semi-major axes range from approximately 10 to 1000 AU.

Similar Systems

There are currently three other systems with a close-in giant planet and a distant outer brown dwarf: Kepler-448 (*113*), WASP-53 (*114*), and WASP-81 (*114*). Kepler-448 is particularly intriguing due the similarities between the giant planets in that system and TOI-201. Both warm Jupiters in the Kepler-448 and TOI-201 systems are moderately eccentric ($e \sim 0.3$) and show transit timing variations due to the outer companion. Both outer companions are highly eccentric ($e \sim 0.6 - 0.65$) and have similar masses and periods (Kepler-448 c has a mass of 22 Jupiter masses and period of 2500 days). Both systems show a significant non-zero mutual inclination between the giant planets, with a mutual inclination of 20 degrees in the Kepler-448 system compared to the 13 degrees between TOI-201 b and c. As with TOI-201, the mechanisms invoked to explain the architecture of Kepler-448 suffer from issues regarding fine-tuning.

Both WASP-53 and WASP-81 contain close-in inner giants, with both inner giants in the systems having orbital periods under 10 days. They do not exhibit detectable TTVs like TOI-201 and Kepler-448, however. Unlike in the case of TOI-201, high-eccentricity migration pathways could explain the WASP-53 and WASP-81 systems, as they both lack a second close-in planet that has to be preserved.

Given how unlikely it is for a single stellar flyby to explain any individual system, it is even more unlikely that all of these systems underwent this process, given the fine-tuning required. In the case of planet-planet scattering, systems can and do form multiple giant planets, often in close proximity to one another. If this occurred, these giant planets were close enough to each other that one was scattered from the system, causing the outer companion to become eccentric. Nonetheless, this mechanism also suffers from a fine-tuning problem.

There is also a potential fifth system, WASP-132, but more observations are needed to determine if there is indeed a brown dwarf present (*115*). WASP-132 contains a small inner planet, a hot Jupiter, an outer giant planet at 2.7 AU, and a long-term RV trend which can be a BD or a stellar companion. If the brown dwarf does exist, then the larger separations between planets could mean scattering never took place, and all of the giant planets that originally formed in the system were preserved. Conversely, the potential brown dwarf in the WASP-132 system could have driven or still be driving vZLK oscillations that sculpted the system's architecture.

Of these systems, TOI-201 has provided the most insight and is poised to continue to do so. It is the only system where the brown dwarf transits, which allows us to measure its radius and opens up opportunities for future observations to study its atmosphere. TOI-201 is also the only system with a confirmed brown dwarf and a super-Earth. It is significantly brighter than the other systems with a J-band magnitude that is 2 magnitudes brighter than the next brightest star. This makes it the target best suited for atmospheric characterization with the James Webb Space Telescope (JWST), as it is 7 times brighter than the other systems in the infrared region of the spectrum that JWST observes in.

Age–radius evolution of the brown dwarf companion

TOI-201 c is one of five confirmed transiting companions at the canonical deuterium fusing mass threshold between giant planets and brown dwarfs of roughly 13 M_J (*116–118*), with the others being HATS-70 b (*119*), TOI-4603 b (*120*), TOI-4987 b (*121*), and TIC 4672985 b (*122*). As with any transiting system, we have the opportunity to examine the radius evolution of the companion given the precise and accurate (when orbiting Sun-like main sequence stars) radius measurements. This is especially important in the case of transiting brown dwarfs as we predict that the radius monotonically contracts with age (*123–125*), meaning that, for isolated brown dwarfs, younger objects will have larger radii than older objects of the same mass. In the case of most transiting brown dwarfs, we must consider the effects of the host star in its energy contribution to the atmosphere of the brown dwarf via irradiation and tidal heating. However, it is clear from the large scaled semi-major axis of ($a/R_\star = 716$) and average incident flux ($\langle F \rangle = 0.15\, S_\oplus$) received by TOI-201 c that we can treat this low-mass brown dwarf as an isolated object with regard to its radius evolution.

This makes TOI-201 c an excellent benchmark system for age–radius evolutionary models for substellar objects. Figure S15 indicates how TOI-201 c's mass, radius, and age compare to brown dwarf and low-mass star models at solar metallicity (*123,126,127*). Interestingly, it lies below where models predict based on the host star's age of approximately 666 Myr. However, it is important to note that there can be significant scatter in brown dwarf models, due to degeneracies between model parameters, including metallicity and the presence, or lack thereof, of clouds (*117, 128*). When we attempt to consider parameters like metallicity, we find that the models better approximate

the radius of the brown dwarf when we assume a metal-poor, cloud-free atmosphere. Even then, the radius of the brown dwarf lies 1.9-σ below the most generous interpretation of the system (i.e. assuming the oldest plausible system age of 2.9 Gyr at the lowest metallicity the models account for). Atmospheric characterization of the brown dwarf would constrain these parameters and help determine why it may be truly smaller than predicted by existing models.

Future Prospects

The dynamical history could be further constrained with a measurement of the system's obliquity relative to the spin axis of the star. While the mutual inclinations between the three known bodies are constrained in this work, the overall obliquity of the system is not. A Rossiter-McLaughlin measurement of TOI-201 b is the most practical way to achieve this. There are multiple transits through the end of 2026 that are observable from sites in Chile and Australia with the capabilities of measuring the expected 30 m/s signal.

A more precise mass measurement of TOI-201 d would reveal whether it is a suitable target for atmospheric characterization with the James Webb Space Telescope (JWST). While its size together with its proximity to the host star likely places it in the rocky planet regime and unlikely to have an atmosphere (*129–131*), it could be a candidate for secondary eclipse spectroscopy to probe the composition and features of its surface. TOI-201 b is a much more promising candidate for transmission spectroscopy, having a Transmission Spectroscopy Metric (TSM) value of 110 which places it in the first quartile for prioritization among giant exoplanets (*132*). Since TOI-201 b is expected to have formed at least one Hill radius interior to TOI-201 c's orbit (i.e. with an initial semi-major axis <3.7 AU), its atmospheric metallicity is predicted to be super-stellar since the planet would likely not have formed in the outer, gas-rich region of the protoplanetary disk (*133*). A measurement of its atmospheric metallicity could test this hypothesis.

Observing a full transit of TOI-201 c will help refine its orbital parameters, specifically its orbital period, and resolve the degeneracy between the transit impact parameter and duration. Additional RV measurements in the next several years, especially in the months preceding the next transit, will help reduce the uncertainty on the transit timing. Afterward, a transit observation can be executed using a combination of ground-based telescopes across the world, including from citizen scientists, as has been done with other single-transit planets (*134*). Despite these challenges, the brown dwarf

is a very promising target for atmospheric characterization. Its long orbital period makes it fairly isolated from its host star and, as a result, should not be inflated like many known transiting brown dwarfs. This makes TOI-201 c an important benchmark for understanding the structure of brown dwarfs, as well as understanding how their radii evolve over time. Atmospheric characterization could reveal important information about its metallicity, which in turn would inform how and where it formed. The existence of a brown dwarf desert near 45 Jupiter masses and differences in the properties between brown dwarfs above and below the desert suggest that high-mass and low-mass brown dwarfs form in different ways (*135*). High-mass brown dwarfs likely form similarly to stars, through fragmentation of the molecular cloud while low-mass brown dwarfs like TOI-201 c are thought to form similarly to planets, through either core accretion or gravitational instability. While it is too close to have formed in-situ through gravitational instability, it is also more massive than what models typically predict can form through core accretion (*136*), although the host star's high metallicity could make it possible (*137*).

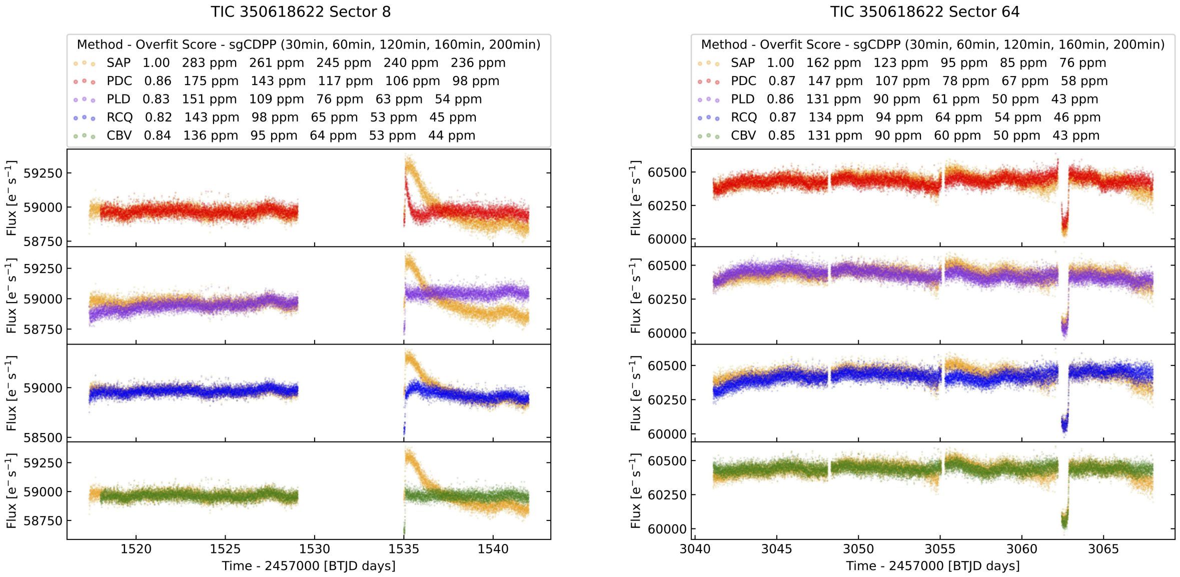

Figure S1: **2-minute TESS SAP light curves plotted against different corrected light curves for Sectors 8 and 64.** The Pixel Level Decorrelation (PLD), Regression Corrector with Quaternions (RCQ), and Cotrending Basis Vectors (CBV) corrected light curves do not exhibit the ramp-like features seen in the PDCSAP light curve.

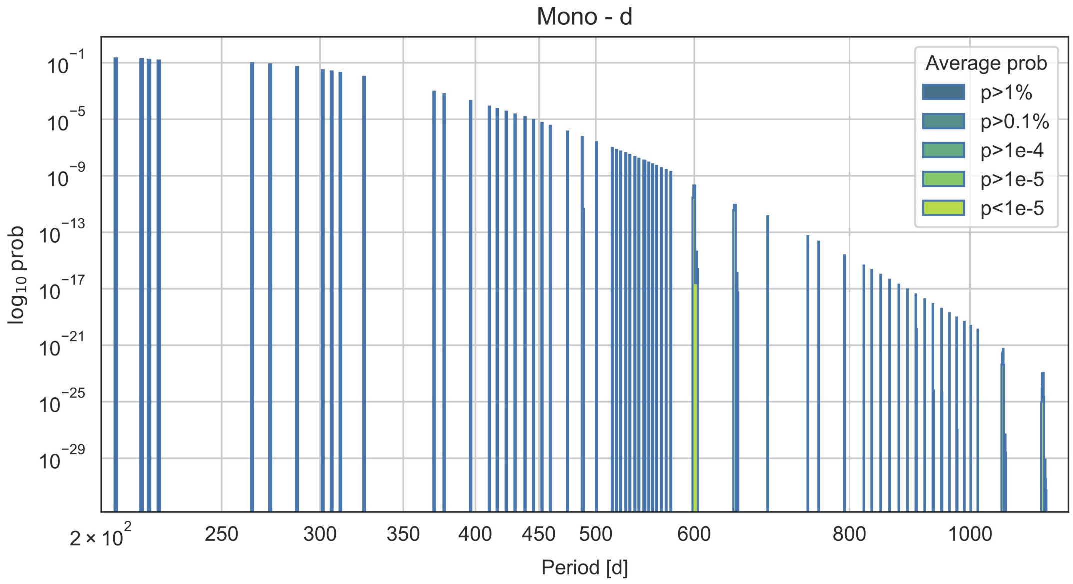

Figure S2: **Periods allowed for TOI-201 c based on TESS photometry alone as determined by `MonoTools`.** Periods below 500 days have uncertainties on the order of a few hours, making them testable from the ground. The marginal probabilities decrease with increasing period as expected from the geometric transit probability.

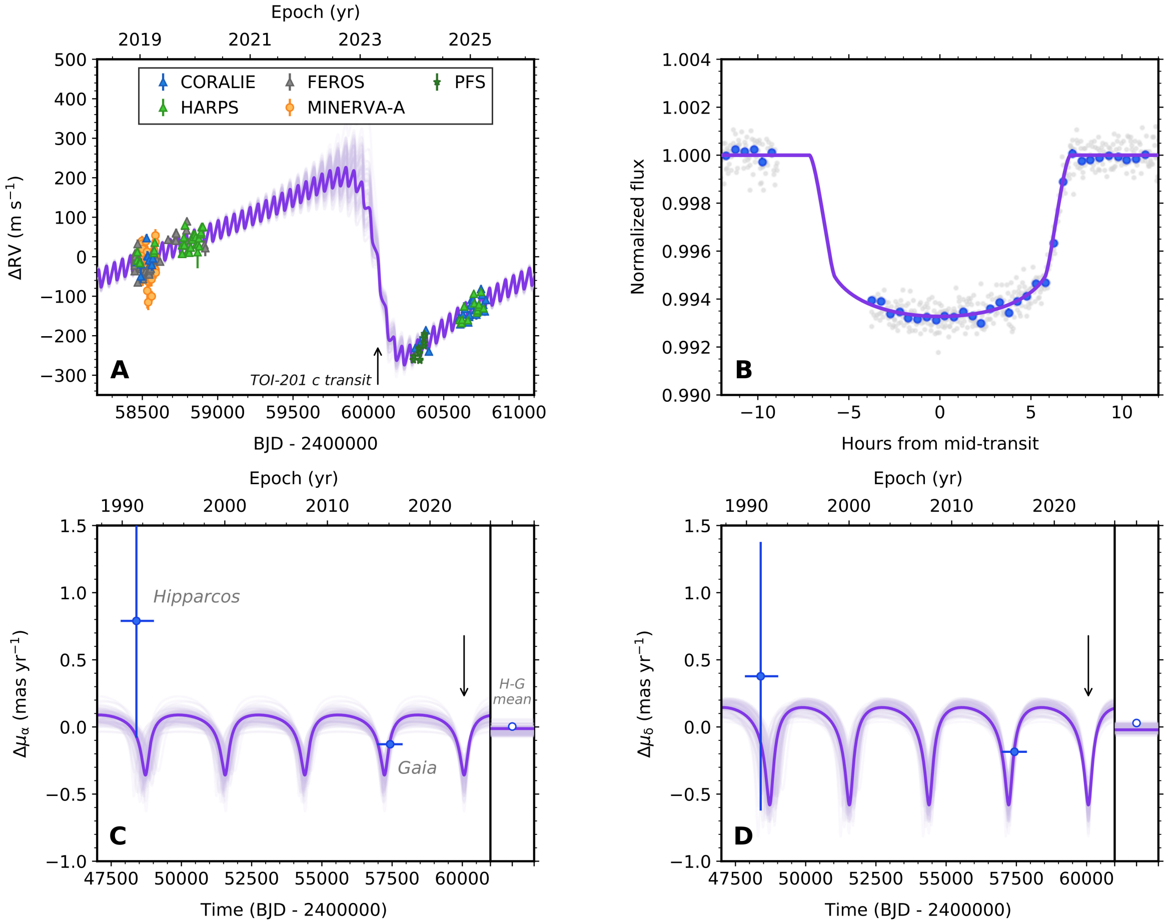

Figure S3: **Joint model of RVs, transits, and *Hipparcos-Gaia* astrometry.** (**A**) RVs and best-fit two-planet RV model. (**B**) TESS photometry and best-fit transit of model TOI-201 c. (**C**) Proper motion in right ascension and best-fit astrometric model for TOI-201 c. The three astrometric points are labelled; the *Hipparcos-Gaia* mean proper motion is shown in the side panel (see (*76*) for further details on the format). (**D**) Proper motion in declination and best-fit astrometric model, formatted as in (**C**). In panels (**A**, **C**, **D**), the epoch of the observed transit of TOI-201 c is marked by an arrow. The *Gaia* observations are coincident with the preceding 2015 periastron passage of TOI-201 c, which explains the significant astrometric acceleration.

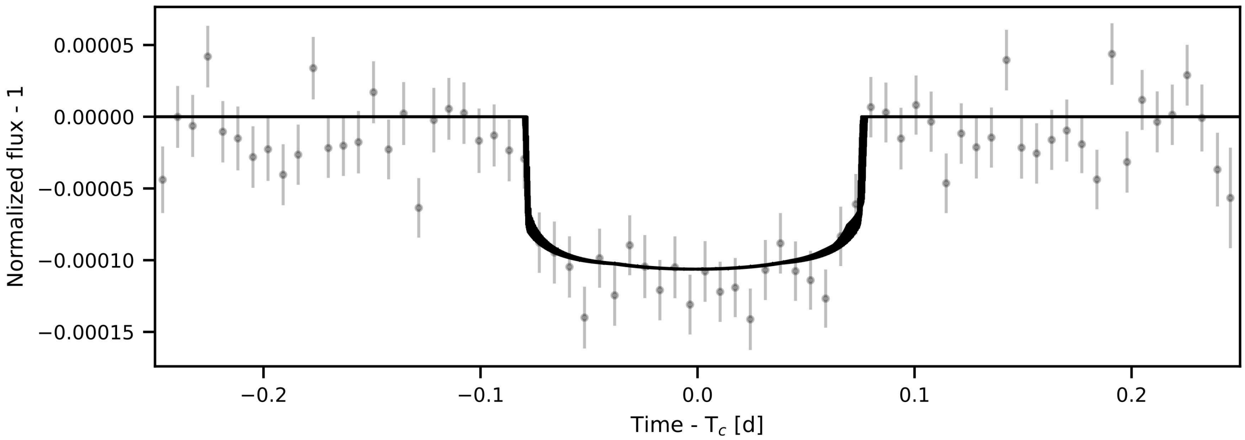

Figure S4: **Phase-folded transits of TOI-201 d in the photodynamical model.** The TESS data is binned for clarity.

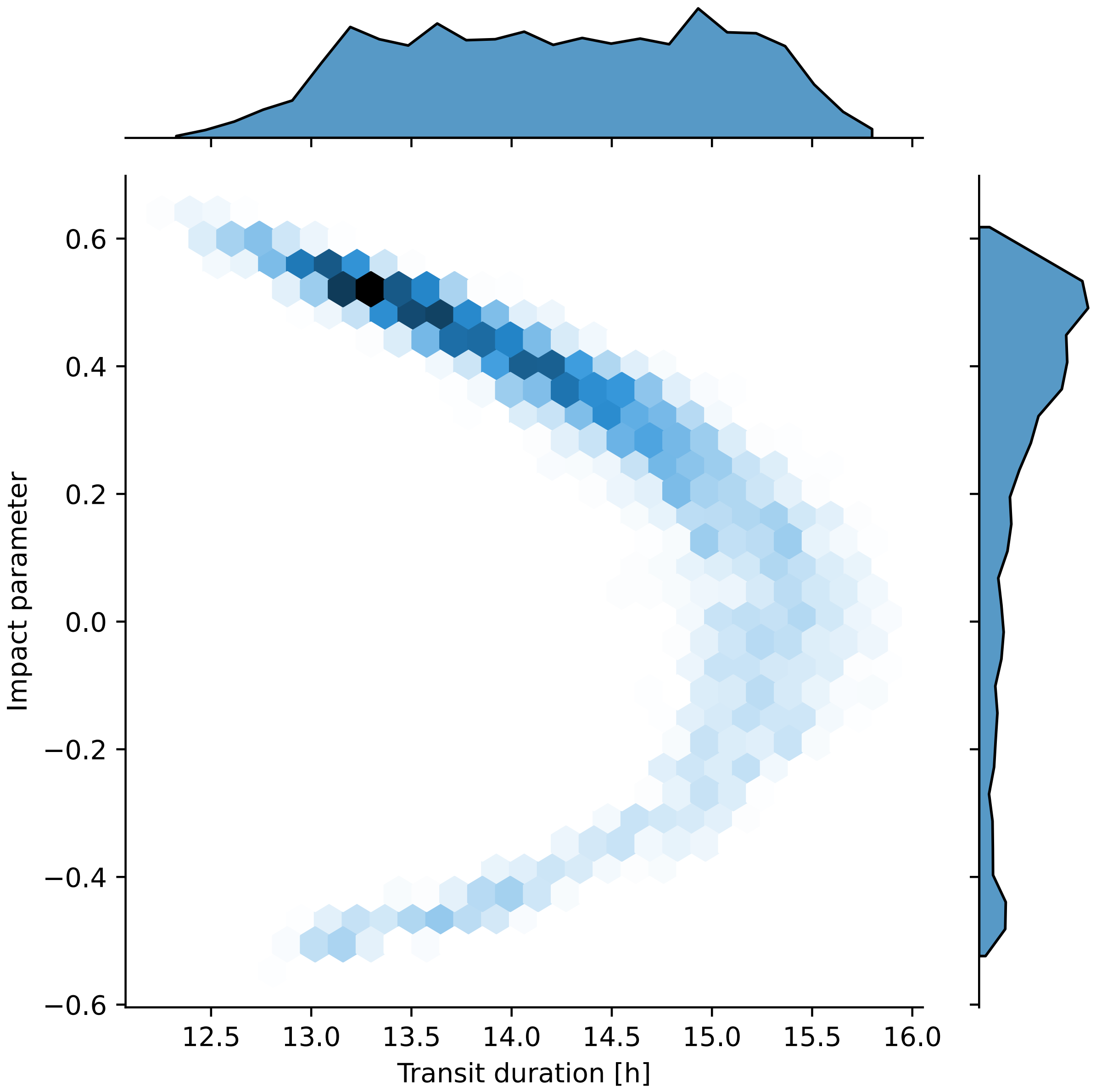

Figure S5: **Duration vs impact parameter for TOI-201 c.** The degeneracy between the two parameters is apparent and is due to the transit not being observed in full.

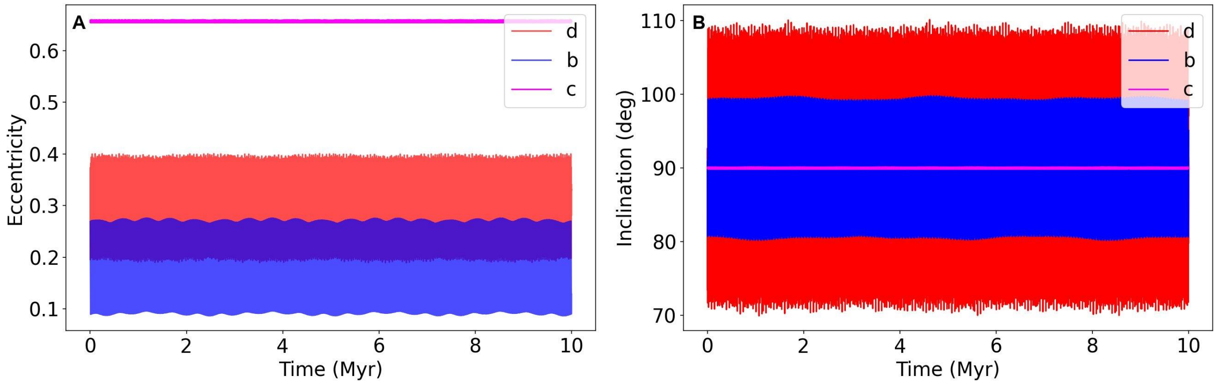

Figure S6: **Dynamical evolution of the TOI-201 system for a 10 Myr integration.** (**A**) The evolution of the eccentricities showing minimal changes for TOI-201 c and larger amplitude oscillations for TOI-201 b and d. (**B**) The evolution of the inclinations showing minimal changes for TOI-201 c, moderate amplitude oscillations for TOI-201 b, and larger amplitude oscillations for TOI-201 d.

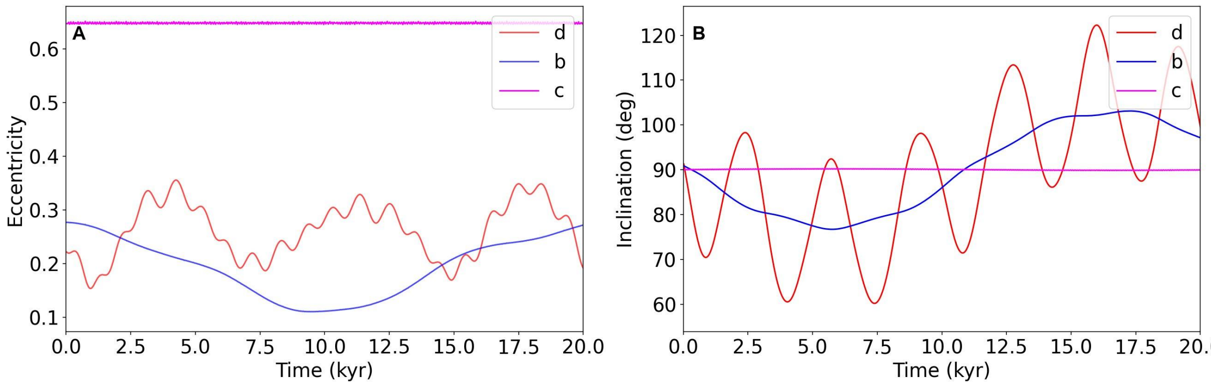

Figure S7: **Secular interactions within the TOI-201 system over 20 kyr.** (**A**) The evolution of the eccentricities showing minimal changes for TOI-201 c, mostly long timescale changes for TOI-201 b, and both long and short timescale changes for TOI-201 d. (**B**) The evolution of the inclinations showing the same patterns as (A).

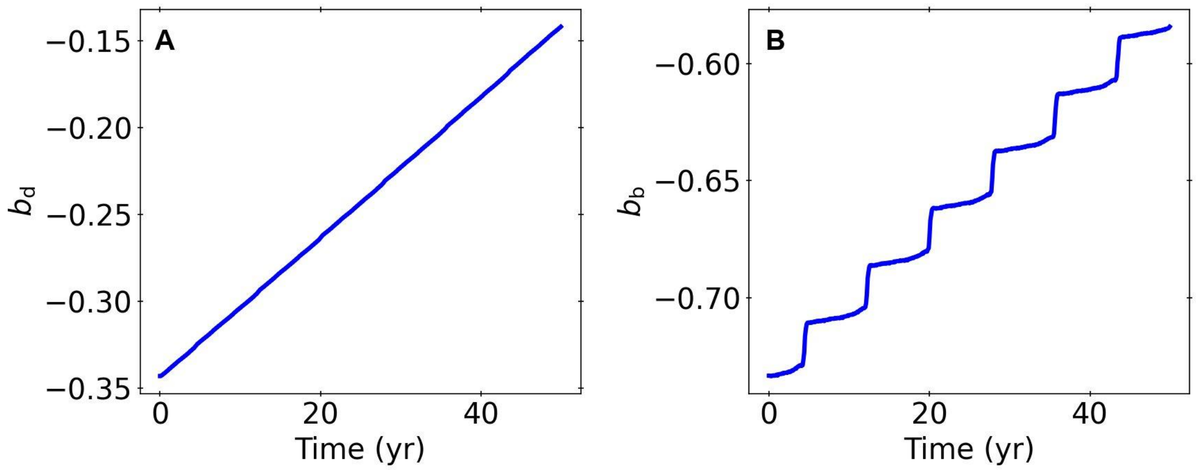

Figure S8: **Short-term dynamics of the impact parameters of the two inner planets.** (**A**) The evolution of the impact parameter for TOI-201 d showing a steady linear change. (**B**) The evolution of the impact parameter for TOI-201 b showing sharp increases after every periastron passage of TOI-201 c.

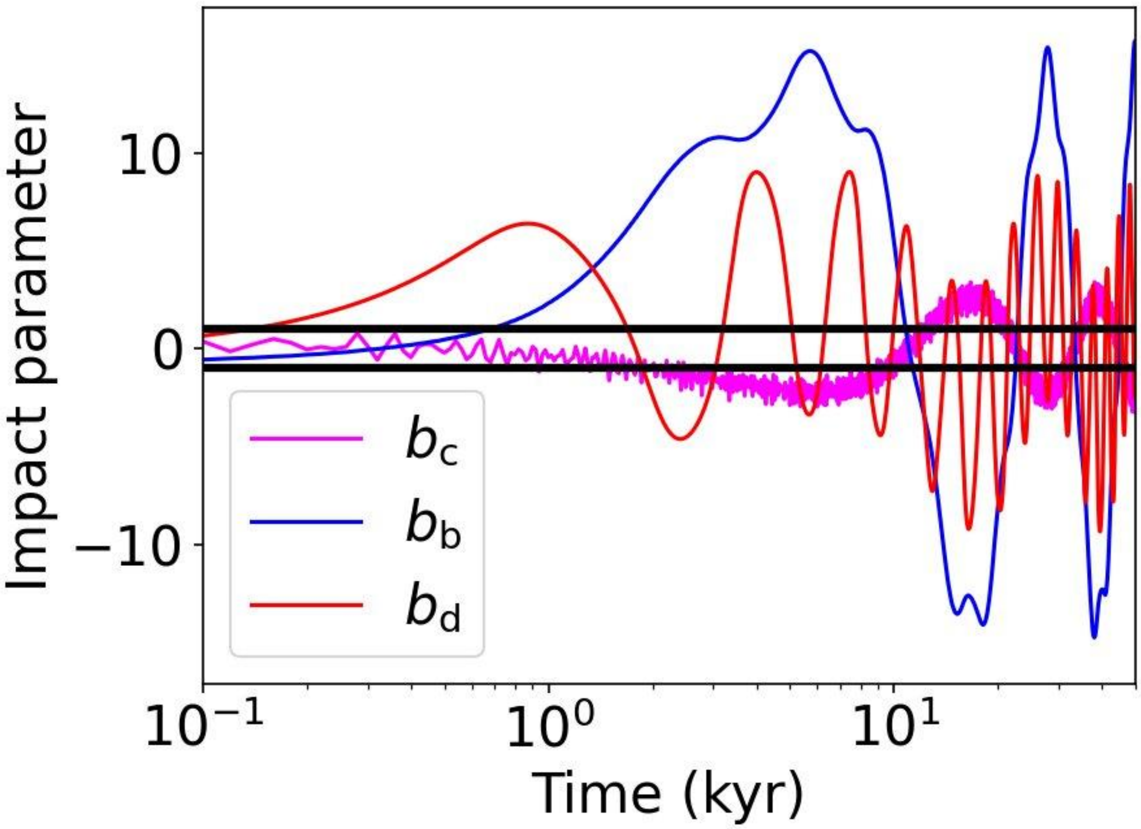

Figure S9: **Dynamics of the impact parameters of TOI-201 b, c, and d over 50 kyr.** All planets are visible via transit observations only when the impact parameters lie between the black horizontal lines, which indicate $b = \pm 1$.

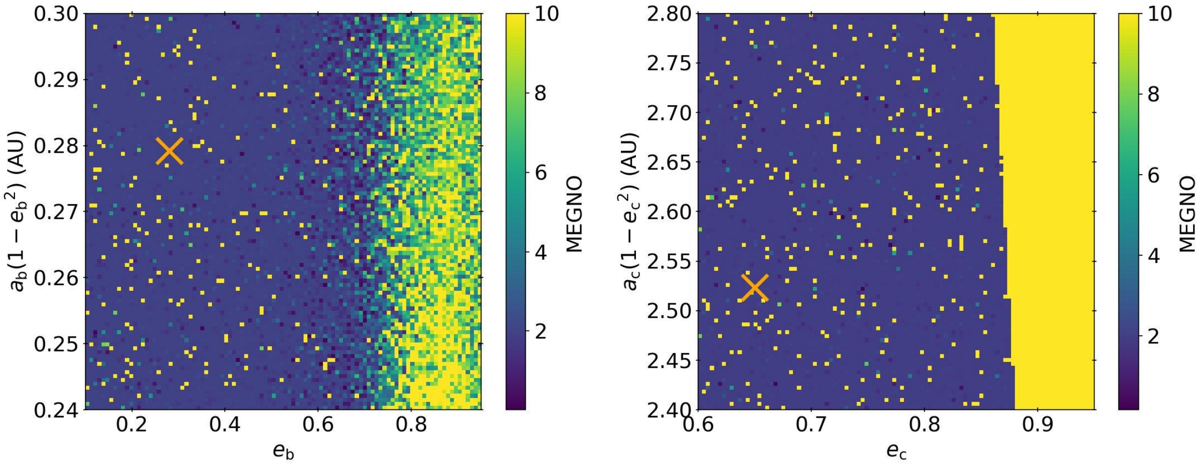

Figure S10: **MEGNO map for TOI-201 b (left) and TOI-201 c (right) for a variety of angular momenta and eccentricities.** The orange marker on each map corresponds to the observed orbital parameters of the system. Since regions where $e \gtrsim 0.9$ result in chaotic evolution and $a(1-e^2)$ is conserved during tidal interactions, we deduce that the system is not consistent with a history of high-eccentricity migration.

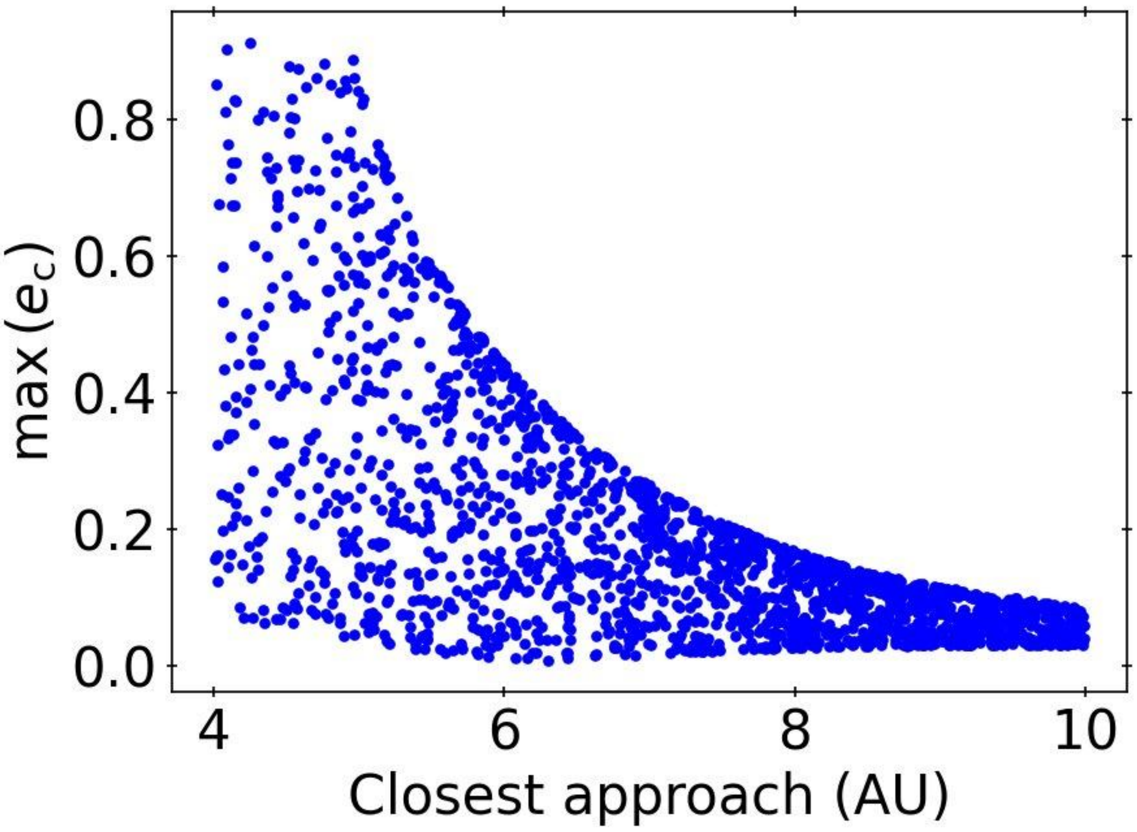

Figure S11: **Maximum eccentricity of TOI-201 c vs. closest approach of perturbing star in stellar fly-by simulations.** The closest approach must be less than 6 AU in order to reproduce the observed eccentricity.

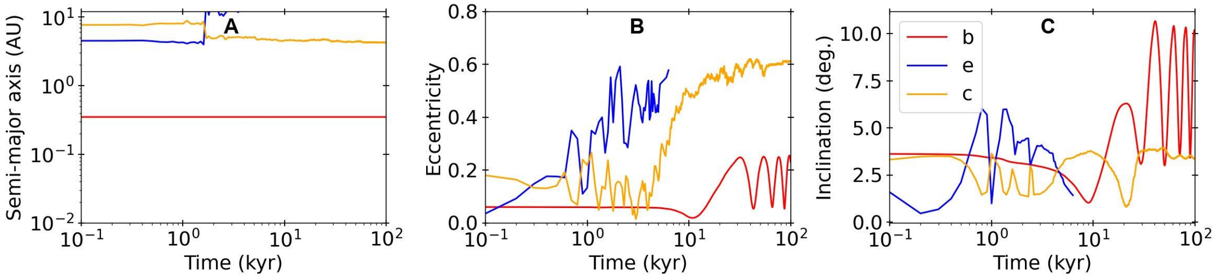

Figure S12: **Dynamical evolution of planet-planet scattering simulation which reproduces the observed architecture of the TOI-201 system.** (**A**) Evolution of the semi-major axes showing the injected planet being ejected and TOI-201 c moving slightly inward. (**B**) Evolution of the eccentricity showing the growth of TOI-201 c's eccentricity and start of oscillations as the injected planet is ejected. (**C**) Evolution of the inclinations.

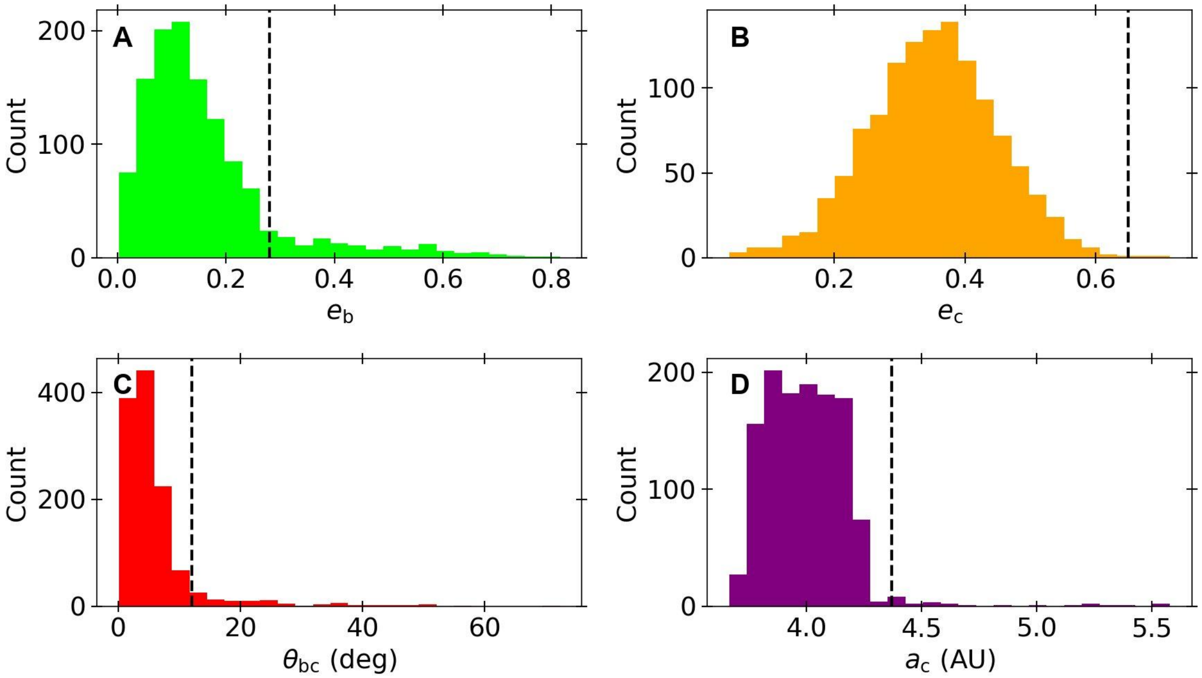

Figure S13: **Distributions of final orbital elements among planet-planet scattering simulations for which the hypothetical TOI-201 e was ejected.** Dashed black lines indicate the observed orbital elements (Table S4). (**A**) Distribution of simulated e_b values showing the present-day value is at the tail end. (**B**) Distribution of simulated e_c values showing the present-day value is at the far end of the distribution. (**C**) Distribution of simulated mutual inclinations. (**D**) Distribution of simulated a_c.

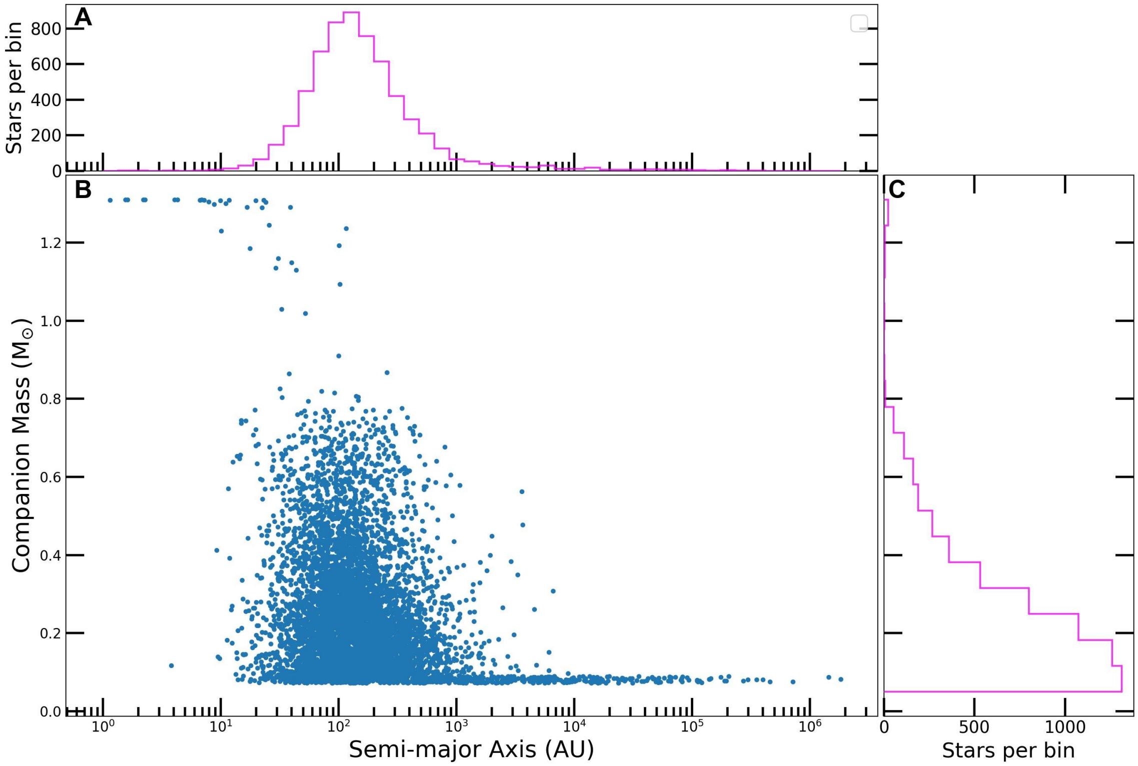

Figure S14: **Stellar companions allowed by the existing data as determined using MOLUSC.** (**A**) Distribution of semi-major axes showing a peak at 100 AU. (**B**) Scatter plot of semi-major axis and companion masses for the allowed companions. (**C**) Distribution of companion masses showing a strong skew towards low masses.

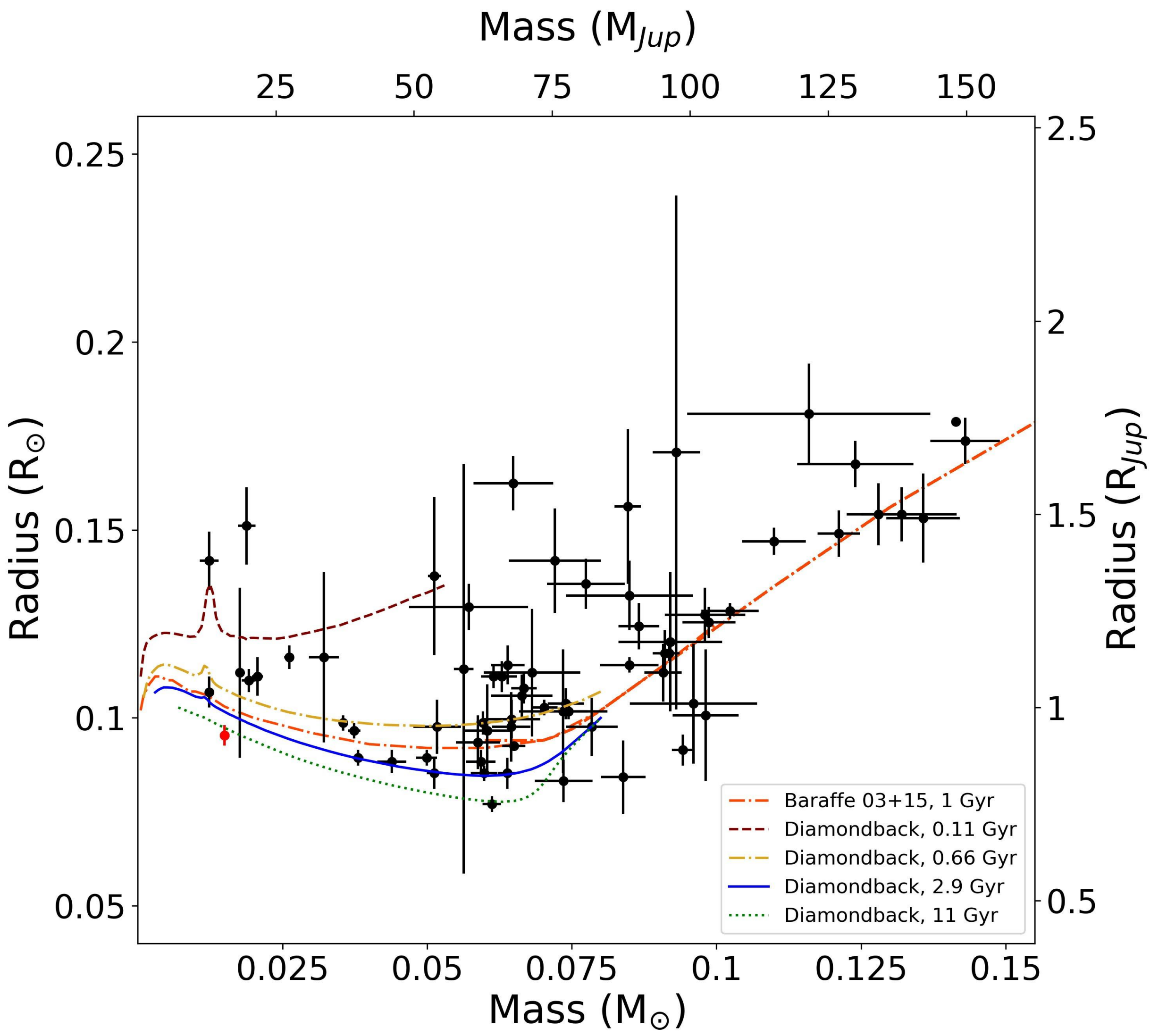

Figure S15: **Mass-radius diagram from transiting brown dwarfs and low-mass stars.** The masses and radii are taken from (*138*) and references therein. The models are for solar metallicity brown dwarfs and low-mass stars of different ages (*125–127*). TOI-201 c is denoted by the red circle and lies well below where predicted for the system's age of 0.66 Gyr.

Table S1: **Observed and derived stellar parameters for TOI-201.** The parameters from this work were obtained from an isochrone fit.

Parameter	Value	Source
TIC	350618622	TICv8 (*139*)
Additional Identifiers	HD 39474; HIP 27515	-
Right Ascension	05:49:36.43	Gaia DR3 (*140*)
Declination	-54:54:37.49	Gaia DR3
μ_α (mas yr^{-1})	8.032 ± 0.018	Gaia DR3
μ_δ (mas yr^{-1})	66.633 ± 0.019	Gaia DR3
Parallax (mas)	8.9141 ± 0.0142	Gaia DR3
B (mag)	9.793 ± 0.29	APASS DR10 (*141*)
V (mag)	9.036 ± 0.020	APASS DR10
G (mag)	8.949289 ± 0.002761	Gaia DR3
B_P (mag)	9.203999 ± 0.002826	Gaia DR3
R_P (mag)	8.532517 ± 0.003806	Gaia DR3
$TESS$ (mag)	8.582 ± 0.006	TICv8
J (mag)	8.103 ± 0.029	2MASS (*142*)
H (mag)	7.923 ± 0.036	2MASS
K_S (mag)	7.846 ± 0.024	2MASS
W_1 (mag)	7.782 ± 0.028	TICv8
W_2 (mag)	7.829 ± 0.020	TICv8
W_3 (mag)	7.834 ± 0.016	TICv8
W_4 (mag)	7.691 ± 0.092	TICv8
$M_\star$ ($M_\odot$)	$1.32^{+0.02}_{-0.04}$	This work
$R_\star$ ($R_\odot$)	1.31 ± 0.01	This work
$\log g$ (log cm s^{-2})	$4.33^{+0.01}_{-0.02}$	This work
$L_\star$ ($L_\odot$)	2.61 ± 0.12	This work
[Fe/H]	0.19 ± 0.06	This work
$T_{\rm eff}$ (K)	6423^{-90}_{+86}	This work
Age (Myr)	666^{+673}_{-442}	This work

Table S2: **Orbital parameters for TOI-201 b and c from our joint model incorporating RVs, transits, and astrometry.** The parameters shown here are consistent with the values we obtain with the full photodynamical model.

Parameter	TOI-201 b	TOI-201 c
Orbital period P (d)	52.979 (fixed)	2834^{+43}_{-38}
Orbital period P (yr)	0.145 (fixed)	$7.76^{+0.12}_{-0.10}$
Semi-amplitude K (m s^{-1})	23.8 ± 1.4	230^{+26}_{-19}
Eccentricity e	$0.287^{+0.038}_{-0.036}$	$0.610^{+0.047}_{-0.041}$
Argument of periastron ω (deg)	$89.3^{+7.5}_{-7.4}$	98.0 ± 6.2
T_p (BJD)	2459965.3 ± 0.6	$2460074.5^{+12.9}_{-9.7}$
T_0 (BJD)	2459965.3944 ± 0.0008	$2460062.579^{+0.016}_{-0.021}$
Impact parameter b	0.73 ± 0.03	$0.41^{+0.13}_{-0.22}$
Radius ratio R_p/R_*	0.07928 ± 0.0009	0.07736 ± 0.0011
Orbital inclination i (deg)	88.82 ± 0.04	$89.916^{+0.044}_{-0.022}$
Longitude of node Ω (deg)	–	212 ± 11
Mass M_p (M_J)	0.505 ± 0.031	$15.4^{+1.0}_{-0.8}$
Radius R_p (R_J)	1.030 ± 0.023	1.005 ± 0.023

Table S3: **Photodynamical model parameters with their priors and posteriors.** $\mathcal{N}(\mu, \sigma)$ denotes a normal prior with a mean μ and standard deviation σ, and $\mathcal{U}(a, b)$ denotes a uniform distribution from a to b. The osculating orbital elements are valid for the reference time $T_{\rm ref}$ = 2458376.052.

Model Parameter	Unit	Prior	Posterior
Stellar parameters			
$R_\star$	$R_\odot$	$\mathcal{N}(1.32, 0.01)$	1.31 ± 0.01
$M_\star$	$M_\odot$	$\mathcal{N}(1.32, 0.03)$	1.33 ± 0.03
q_1		$\mathcal{U}(0, 1)$	0.23 ± 0.03
q_2		$\mathcal{U}(0, 1)$	< 0.36
RV parameters			
$\gamma_{\rm MINERVA}$	$\rm m\,s^{-1}$	$\mathcal{N}(0, 50)$	-3 ± 5
$\gamma_{\rm HARPS}$	$\rm m\,s^{-1}$	$\mathcal{N}(16700, 50)$	16731 ± 2
$\gamma_{\rm CORALIE}$	$\rm m\,s^{-1}$	$\mathcal{N}(16700, 50)$	16846 ± 3
$\gamma_{\rm FEROS}$	$\rm m\,s^{-1}$	$\mathcal{N}(16865, 50)$	16866 ± 4
$\gamma_{\rm PFS}$	$\rm m\,s^{-1}$	$\mathcal{N}(250, 50)$	232 ± 4
$\log_{10} \sigma_{\rm MINERVA}$	$\log_{10}$ $\rm m\,s^{-1}$	$\mathcal{N}(-1, 1)$	1.53 ± 0.05
$\log_{10} \sigma_{\rm HARPS}$	$\log_{10}$ $\rm m\,s^{-1}$	$\mathcal{N}(-3, 0.1)$	-3.0 ± 0.1
$\log_{10} \sigma_{\rm CORALIE}$	$\log_{10}$ $\rm m\,s^{-1}$	$\mathcal{N}(-1, 1)$	-0.9 ± 0.8
$\log_{10} \sigma_{\rm FEROS}$	$\log_{10}$ $\rm m\,s^{-1}$	$\mathcal{N}(-1, 1)$	1.28 ± 0.05
$\log_{10} \sigma_{\rm PFS}$	$\log_{10}$ $\rm m\,s^{-1}$	$\mathcal{N}(-1, 1)$	0.82 ± 0.09
TOI-201 d			
P_d	days	$\mathcal{N}(5.849, 0.005)$	5.8489 ± 0.0001
$T_{0,\rm d}$	BJD	$\mathcal{N}(2458374.033, 0.003)$	2458374.032 ± 0.003
$\log_{10} M_{\rm p,d}$	$\log_{10}$ $M_\odot$	$\mathcal{N}(-5.07, 0.15)$	-4.8 ± 0.1
$R_{\rm p,d}/R_\star$		$\mathcal{N}(0.01, 0.001)$	0.0097 ± 0.0005
$\sqrt{e_{\rm d}} \cos \omega_{\rm d}$		$\mathcal{U}(-1, 1)$	-0.06 ± 0.09
$\sqrt{e_{\rm d}} \sin \omega_{\rm d}$		$\mathcal{U}(-1, 1)$	0.47 ± 0.09
$b_{\rm d}$		$\mathcal{U}(-1, 1)$	-0.1 ± 0.3
$\Omega_{\rm d}$	rad	$\mathcal{U}(0.5\pi, 1.5\pi)$	3.1 ± 0.4
TOI-201 b			
$P_{\rm b}$	days	$\mathcal{N}(52.980, 0.005)$	52.9786 ± 0.0001
$T_{0,\rm b}$	BJD	$\mathcal{N}(2458376.0520, 0.0002)$	2458376.0521 ± 0.0002
$\log_{10} M_{\rm p,b}$	$\log_{10}$ $M_\odot$	$\mathcal{U}(-4.0, -2.9)$	-3.31 ± 0.01
$R_{\rm p,b}/R_\star$		$\mathcal{N}(0.07, 0.003)$	0.0798 ± 0.0004
$\sqrt{e_{\rm b}} \cos \omega_{\rm b}$		$\mathcal{U}(-1, 1)$	0.522 ± 0.008
$\sqrt{e_{\rm b}} \sin \omega_{\rm b}$		$\mathcal{U}(-1, 1)$	0.06 ± 0.02
$b_{\rm b}$		$\mathcal{U}(-1, 1)$	-0.741 ± 0.006
$\Omega_{\rm b}$	rad	$\mathcal{U}(0.5\pi, 1.5\pi)$	3.5 ± 0.2
TOI-201 c			
$P_{\rm c}$	days	$\mathcal{U}(2300, 3500)$	2890 ± 20
$T_{0,\rm c}$	BJD	$\mathcal{N}(2460062.6, 0.1)$	2460062.59 ± 0.02
$\log_{10} M_{\rm p,c}$	$\log_{10}$ $M_\odot$	$\mathcal{U}(-2.5, -1.2)$	-1.824 ± 0.009
$R_{\rm p,c}/R_\star$		$\mathcal{N}(0.07, 0.003)$	0.073 ± 0.002
$\sqrt{e_{\rm c}} \cos \omega_{\rm c}$		$\mathcal{U}(-1, 1)$	0.800 ± 0.0005
$\sqrt{e_{\rm c}} \sin \omega_{\rm c}$		$\mathcal{U}(-1, 1)$	-0.09 ± 0.03
$b_{\rm c}$		$\mathcal{U}(-1, 1)$	0.4 ± 0.3
$\Omega_{\rm c}$	rad	$\mathcal{N}(3.70, 0.19)$	3.7 ± 0.2

Table S4: **Photodynamical final posterior parameters.** The osculating orbital elements are valid for the reference time $T_{\mathrm{ref}} = 2458376.052$.

Parameter	Unit	TOI-201 d	TOI-201 b	TOI-201 c
P	days	5.8489 ± 0.0001	52.9786 ± 0.0001	2890 ± 20
T_0	BJD-2458000	374.032 ± 0.003	376.0521 ± 0.0002	2062.59 ± 0.02
M_{p}	$M_{\oplus}$	5.8 ± 2	164 ± 5	4990 ± 100
R_{p}	$R_{\oplus}$	1.39 ± 0.07	11.4 ± 0.1	10.4 ± 0.3
ρ_{p}	$\mathrm{g\,cm^{-3}}$	11 ± 4	0.61 ± 0.02	24 ± 2
T_{14}	h	3.76 ± 0.08	4.13 ± 0.01	13.1 ± 0.8
e		0.3 ± 0.1	0.275 ± 0.009	0.651 ± 0.006
ω	°	340 ± 80	83 ± 2	96 ± 2
i	°	91.7 ± 1.6	91.18 ± 0.02	$89.92^{+0.10}_{-0.04}$
Ω	°	175 ± 20	198 ± 10	211 ± 11
$a/R_{\star}$		11.4 ± 0.1	49.7 ± 0.4	716 ± 8
a	AU	0.0698 ± 0.0005	0.303 ± 0.002	4.37 ± 0.04

Table S5: **New time series radial velocities from CORALIE.**

Time (BJD-2457000)	RV (m s^{-1})	RV Uncertainty (m s^{-1})	Instrument
3311.6364	16610.90	9.16	CORALIE
3344.6467	16622.50	18.63	CORALIE
3362.6982	16622.35	15.39	CORALIE
3381.5481	16655.99	7.90	CORALIE
3401.5122	16601.94	7.72	CORALIE
3602.7809	16686.58	8.57	CORALIE
3609.7397	16670.91	8.11	CORALIE
3616.7343	16674.18	9.68	CORALIE
3623.7228	16679.83	7.60	CORALIE
3627.5930	16695.77	8.41	CORALIE
3634.6395	16715.16	11.59	CORALIE
3640.7489	16710.02	8.69	CORALIE
3662.6176	16675.55	9.74	CORALIE
3668.7606	16689.79	8.77	CORALIE
3670.5819	16694.50	10.12	CORALIE
3678.6534	16718.74	9.23	CORALIE
3685.5400	16733.62	11.95	CORALIE
3694.7396	16732.99	10.51	CORALIE
3709.5339	16706.52	10.09	CORALIE
3716.6576	16695.25	12.68	CORALIE
3723.5855	16714.22	9.18	CORALIE
3730.5526	16707.76	10.50	CORALIE
3747.5246	16760.30	9.73	CORALIE
3754.5644	16745.53	8.76	CORALIE
3762.5604	16720.02	10.22	CORALIE
3772.5339	16703.63	11.30	CORALIE
3779.4784	16732.45	9.72	CORALIE

Table S6: New time series radial velocities from HARPS.

Time (BJD-2457000)	RV (m s^{-1})	RV Uncertainty (m s^{-1})	Instrument
3603.6999	16569.70	9.25	HARPS
3615.8744	16561.90	5.02	HARPS
3618.8081	16562.23	6.71	HARPS
3621.6992	16570.73	4.51	HARPS
3635.8250	16604.25	5.87	HARPS
3660.5947	16569.28	4.36	HARPS
3699.7400	16636.26	5.21	HARPS
3702.6655	16612.15	3.82	HARPS
3720.5526	16588.90	4.93	HARPS
3724.6358	16594.12	3.86	HARPS
3731.5862	16603.57	3.83	HARPS
3733.5648	16609.06	4.73	HARPS
3747.6424	16641.06	4.25	HARPS
3764.5931	16599.30	5.20	HARPS

Table S7: Time series radial velocities from PFS.

Time (BJD-2457000)	RV (m s^{-1})	RV Uncertainty (m s^{-1})	Instrument
3298.6407	-28.42	2.56	PFS
3298.7281	-26.93	2.21	PFS
3301.6846	-18.07	2.51	PFS
3301.7776	-19.36	2.38	PFS
3334.5868	-0.30	1.84	PFS
3334.6753	1.94	1.58	PFS
3336.5880	0.32	1.94	PFS
3336.6665	0.00	1.67	PFS
3338.6077	-12.25	1.58	PFS
3338.6739	-9.47	1.55	PFS
3341.5710	-29.83	1.53	PFS
3341.6600	-26.93	1.61	PFS
3369.5364	27.97	2.00	PFS
3369.5424	27.54	2.04	PFS
3370.5238	14.63	2.07	PFS
3370.5297	7.02	1.97	PFS
3370.6154	15.11	2.04	PFS
3372.5267	38.40	1.70	PFS
3372.5978	33.18	1.75	PFS

Caption for Data S1. CORALIE radial velocity time series. Machine readable file of CORALIE radial velocity time series as Barycentric Julian Dates (BJD), radial velocities, and associated uncertainties in m s^{-1}.

Caption for Data S2. FEROS radial velocity time series. Machine readable file of FEROS radial velocity time series as Barycentric Julian Dates (BJD), radial velocities, and associated uncertainties in m s^{-1}.

Caption for Data S3. HARPS radial velocity time series. Machine readable file of HARPS radial velocity time series as Barycentric Julian Dates (BJD), radial velocities, and associated uncertainties in m s^{-1}.

Caption for Data S4. MINERVA-Australis radial velocity time series. Machine readable file of MINERVA-Australis radial velocity time series as Barycentric Julian Dates (BJD), radial velocities, and associated uncertainties in m s^{-1}.

Caption for Data S5. PFS radial velocity time series. Machine readable file of PFS radial velocity time series as Barycentric Julian Dates (BJD), radial velocities, and associated uncertainties in m s^{-1}.

REFERENCES

1. M. J. Hobson, R. Brahm, A. Jordán, N. Espinoza, D. Kossakowski, T. Henning, F. Rojas, M. Schlecker, P. Sarkis, T. Trifonov, D. Thorngren, A. Binnenfeld, S. Shahaf, S. Zucker, G. R. Ricker, D. W. Latham, S. Seager, J. N. Winn, J. M. Jenkins, B. Addison, F. Bouchy, B. P. Bowler, J. T. Briegal, E. M. Bryant, K. A. Collins, T. Daylan, N. Grieves, J. Horner, C. Huang, S. R. Kane, J. Kielkopf, B. McLean, M. W. Mengel, L. D. Nielsen, J. Okumura, M. Jones, P. Plavchan, A. Shporer, A. M. S. Smith, R. Tilbrook, C. G. Tinney, J. D. Twicken, S. Udry, N. Unger, R. West, R. A. Wittenmyer, B. Wohler, P. Torres, D. J. Wright, A transiting warm giant planet around the young active star TOI-201. *Astron. J.* **161**, 235 (2021).

2. R. I. Dawson, R. A. Murray-Clay, J. A. Johnson, The photoeccentric effect and Proto-hot Jupiters. III. A paucity of Proto-hot Jupiters on super-eccentric orbits. *Astrophys. J.* **798**, 66 (2015).

3. C. Huang, Y. Wu, A. H. M. J. Triaud, Warm Jupiters are less lonely than hot Jupiters: Close neighbors. *Astrophys. J.* **825**, 98 (2016).

4. D.-H. Wu, M. Rice, S. Wang, Evidence for hidden nearby companions to hot Jupiters. *Astron. J.* **165**, 171 (2023).

5. A. C. Boley, A. P. Granados Contreras, B. Gladman, The in situ formation of giant planets at short orbital periods. *Astrophys. J. Lett.* **817**, L17 (2016).

6. K. J. Walsh, A. Morbidelli, S. N. Raymond, D. P. O’Brien, A. M. Mandell, A low mass for Mars from Jupiter’s early gas-driven migration. *Nature* **475**, 206–209 (2011).

7. T. Hallatt, E. J. Lee, Can large-scale migration explain the giant planet occurrence rate? *Astrophys. J.* **904**, 134 (2020).

8. E. B. Ford, F. A. Rasio, Origins of eccentric extrasolar planets: Testing the planet-planet scattering model. *Astrophys. J.* **686**, 621–636 (2008).

9. C. Petrovich, Hot jupiters from coplanar high-eccentricity migration. *Astrophys. J.* **805**, 75 (2015).

10. M. Vick, D. Lai, K. R. Anderson, Chaotic tides in migrating gas giants: Forming hot and transient warm Jupiters via Lidov–Kozai migration. *Mon. Not. R. Astron. Soc.* **484**, 5645–5668 (2019).

11. M. L. Bryan, H. A. Knutson, A. W. Howard, H. Ngo, K. Batygin, J. R. Crepp, B. J. Fulton, S. Hinkley, H. Isaacson, J. A. Johnson, Statistics of long period gas giant planets in known planetary systems. *Astrophys. J.* **821**, 89 (2016).

12. R. I. Dawson, J. A. Johnson, Origins of hot Jupiters. *Annu. Rev. Astron. Astrophys.* **56**, 175–221 (2018).

13. N. M. Guerrero, S. Seager, C. X. Huang, A. Vanderburg, A. G. Soto, I. Mireles, K. Hesse, W. Fong, A. Glidden, A. Shporer, D. W. Latham, K. A. Collins, S. N. Quinn, J. Burt, D. Dragomir, I. Crossfield, R. Vanderspek, M. Fausnaugh, C. J. Burke, G. Ricker, T. Daylan, Z. Essack, M. N. Günther, H. P. Osborn, J. Pepper, P. Rowden, L. Sha, S. Villanueva Jr., D. A. Yahalomi, L. Yu, S. Ballard, N. M. Batalha, D. Berardo, A. Chontos, J. A. Dittmann, G. A. Esquerdo, T. Mikal-Evans, R. Jayaraman, A. Krishnamurthy, D. R. Louie, N. Mehrle, P. Niraula, B. V. Rackham, J. E. Rodriguez, S. J. L. Rowden, C. Sousa-Silva, D. Watanabe, I. Wong, Z. Zhan, G. Zivanovic, J. L. Christiansen, D. R. Ciardi, M. A. Swain, M. B. Lund, S. E. Mullally, S. W. Fleming, D. R. Rodriguez, P. T. Boyd, E. V. Quintana, T. Barclay, K. D. Colón, S. A. Rinehart, J. E. Schlieder, M. Clampin, J. M. Jenkins, J. D. Twicken, D. A. Caldwell, J. L. Coughlin, C. Henze, J. J. Lissauer, R. L. Morris, M. E. Rose, J. C. Smith, P. Tenenbaum, E. B. Ting, B. Wohler, G. Á. Bakos, J. L. Bean, Z. K. Berta-Thompson, A. Bieryla, L. G. Bouma, L. A. Buchhave, N. Butler, D. Charbonneau, J. P. Doty, J. Ge, M. J. Holman, A. W. Howard, L. Kaltenegger, S. R. Kane, H. Kjeldsen, L. Kreidberg, D. N. C. Lin, C. Minsky, N. Narita, M. Paegert, A. Pál, E. Palle, D. D. Sasselov, A. Spencer, A. Sozzetti, K. G. Stassun, G. Torres, S. Udry, J. N. Winn, The TESS objects of interest catalog from the TESS prime mission. *Astrophys. J. Suppl. Ser.* **254**, 39 (2021).

14. S. Giacalone, C. D. Dressing, triceratops: Candidate exoplanet rating tool (2020).

15. G. Maciejewski, W. Łoboda, A transiting giant on a 7.7-year orbit revealed by TTVs in the TOI-201 system. arXiv:2507.11504 [astro-ph.EP] (2025).

16. S. Seager, G. Mallén-Ornelas, A unique solution of planet and star parameters from an extrasolar planet transit light curve. *Astrophys. J.* **585**, 1038–1055 (2003).

17. G. Dransfield, D. Mékarnia, A. H. M. J. Triaud, T. Guillot, L. Abe, L. J. García, M. Timmermans, N. Crouzet, F.-X. Schmider, A. Agabi, O. Suarez, P. Bendjoya, M. N. Guenther, O. Lai, B. Merin, P. Stee, Observation scheduling and automatic data reduction for the Antarctic Telescope, ASTEP+, in *Observatory Operations: Strategies, Processes, and Systems IX*, D. S. Adler, R. L. Seaman, C. R. Benn, Eds., vol. 12186 of *Society of Photo-Optical Instrumentation Engineers (SPIE) Conference Series* (2022), p. 121861F.

18. F.-X. Schmider, L. Abe, A. Agabi, P. Bendjoya, N. Crouzet, G. Dransfield, T. Guillot, O. Lai, D. Mekarnia, O. Suarez, A. H. M. J. Triaud, P. Stee, M. N. Günther, D. Breeveld, S. Blommaert, Observing exoplanets from Antarctica in two colours: Set-up and operation of ASTEP+, in *Ground-based and Airborne Telescopes IX*, H. K. Marshall, J. Spyromilio, T. Usuda, Eds., vol. 12182 of *Society of Photo-Optical Instrumentation Engineers (SPIE) Conference Series* (2022), p. 121822O.

19. P. Kervella, F. Arenou, F. Mignard, F. Thévenin, Stellar and substellar companions of nearby stars from Gaia DR2. Binarity from proper motion anomaly. *Astron. Astrophys.* **623**, A72 (2019).

20. T. D. Brandt, The *Hipparcos–Gaia* catalog of accelerations. *Astrophys. J. Suppl. Ser.* **239**, 31 (2018).

21. T. D. Brandt, The Hipparcos–Gaia catalog of accelerations: Gaia EDR3 edition. *Astrophys. J. Suppl. Ser.* **254**, 42 (2021).

22. D. S. Spiegel, A. Burrows, J. A. Milsom, The Deuterium-burning mass limit for brown dwarfs and giant planets. *Astrophys. J.* **727**, 57 (2011).

23. M. M. Romanova, A. V. Koldoba, G. V. Ustyugova, D. Lai, R. V. Lovelace, Eccentricity growth of massive planets inside cavities of protoplanetary discs. *Mon. Not. R. Astron. Soc.* **523**, 2832–2849 (2023).

24. S. Chatterjee, E. B. Ford, S. Matsumura, F. A. Rasio, Dynamical outcomes of planet-planet scattering. *Astrophys. J.* **686**, 580–602 (2008).

25. D. Carrera, S. N. Raymond, M. B. Davies, Planet–planet scattering as the source of the highest eccentricity exoplanets. *Astron. Astrophys.* **629**, L7 (2019).

26. G. Takeda, F. A. Rasio, High orbital eccentricities of extrasolar planets induced by the Kozai mechanism. *Astrophys. J.* **627**, 1001–1010 (2005).

27. D. Fabrycky, S. Tremaine, Shrinking binary and planetary orbits by Kozai cycles with tidal friction. *Astrophys. J.* **669**, 1298–1315 (2007).

28. T. Ito, K. Ohtsuka, The Lidov-Kozai oscillation and Hugo von Zeipel. *Monogr. Environ. Earth Planets* **7**, 1–113 (2019).

29. H. Rein, S.-F. Liu, REBOUND: An open-source multi-purpose N-body code for collisional dynamics. *Astron. Astrophys.* **537**, A128 (2012).

30. H. Rein, D. Tamayo, WHFAST: A fast and unbiased implementation of a symplectic Wisdom–Holman integrator for long-term gravitational simulations. *Mon. Not. R. Astron. Soc.* **452**, 376–388 (2015).

31. J. Li, D. Lai, Resonant excitation of planetary eccentricity due to a dispersing eccentric protoplanetary disk: A new mechanism of generating large planetary eccentricities. *Astrophys. J.* **956**, 17 (2023).

32. M. Jurić, S. Tremaine, Dynamical origin of extrasolar planet eccentricity distribution. *Astrophys. J.* **686**, 603–620 (2008).

33. R. Frelikh, H. Jang, R. A. Murray-Clay, C. Petrovich, Signatures of a planet–planet impacts phase in exoplanetary systems hosting giant planets. *Astrophys. J. Lett.* **884**, L47 (2019).

34. D. Tamayo, H. Rein, P. Shi, D. M. Hernandez, REBOUNDx: A library for adding conservative and dissipative forces to otherwise symplectic N-body integrations. *Mon. Not. R. Astron. Soc.* **491**, 2885–2901 (2020).

35. J. M. Jenkins, J. D. Twickenb, S. M. Cauliffc, J. Campbellc, D. Sanderfera, D. Lungd, M. Mansouri-Samanie, F. Girouardf, P. Tenenbaumb, T. Klause, J. C. Smithb, D. A. Caldwellb, A. Dean Chacond, C. Henzea, C. Heigesg, D. W. Lathamh, E. Morgani, D. Swadej, S. Rinehartk, R. Vanderspeki, The TESS Science Processing Operations Center, in *Software and Cyberinfrastructure for Astronomy IV*, vol. 9913 of (2016), p. 99133E.

36. J. M. Jenkins, The impact of solar-like variability on the detectability of transiting terrestrial planets. *Astrophys. J.* **575**, 493–505 (2002).

37. J. M. Jenkins, H. Chandrasekarana, b, Sean D. Mc Cauliffc, D. A. Caldwella, P. Tenenbauma, J. Lia, T. C. Klausc, M. T. Coted, C. Middourc, Transiting planet search in the Kepler pipeline, in *Software and Cyberinfrastructure for Astronomy*, N. M. Radziwill, A. Bridger, Eds., vol. 7740 of *Society of Photo-Optical Instrumentation Engineers (SPIE) Conference Series* (2010), p. 77400D.

38. J. M. Jenkins, P. Tenenbaum, S. Seader, C. J. Burke, S. D. McCauliff, J. C. Smith, J. D. Twicken, H. Chandrasekaran, Kepler Data Processing Handbook: Transiting Planet Search, Kepler Science Document KSCI-19081-003 (2020).

39. J. Li, P. Tenenbaum, J. D. Twicken, C. J. Burke, J. M. Jenkins, E. V. Quintana, J. F. Rowe, S. E. Seader, *Kepler* data validation II – Transit model fitting and multiple-planet search. *Publ. Astron. Soc. Pac.* **131**, 024506 (2019).

40. J. D. Twicken, J. Catanzarite, B. Clarke, F. Girouard, J. Jenkins, T. Klaus, J. Li, S. McCauliff, S. Seader, P. Tenenbaum, B. Wohler, S. Bryson, C. Burke, D. Caldwell, M. Haas, C. Henze, D.

Sanderfer, Kepler data validation I — Architecture, diagnostic tests, and data products for vetting transiting planet candidates. *Publ. Astron. Soc. Pac.* **130**, 064502 (2018).

41. C. X. Huang, A. Vanderburg, A. Pál, L. Sha, L. Yu, W. Fong, M. Fausnaugh, A. Shporer, N. Guerrero, R. Vanderspek, G. Ricker, Photometry of 10 million stars from the first two years of TESS full frame images: Part I. *Res. Notes Am. Astron. Soc.* **4**, 204 (2020).

42. C. X. Huang, A. Vanderburg, A. Pál, L. Sha, L. Yu, W. Fong, M. Fausnaugh, A. Shporer, N. Guerrero, R. Vanderspek, G. Ricker, Photometry of 10 million stars from the first two years of TESS full frame images: Part II. *Res. Notes Am. Astron. Soc.* **4**, 206 (2020).

43. J. C. Smith, M. C. Stumpe, J. E. Van Cleve, J. M. Jenkins, T. S. Barclay, M. N. Fanelli, F. R. Girouard, J. J. Kolodziejczak, S. D. McCauliff, R. L. Morris, J. D. Twicken, *Kepler* presearch data conditioning II – A Bayesian approach to systematic error correction. *Publ. Astron. Soc. Pac.* **124**, 1000 (2012).

44. M. C. Stumpe, J. C. Smith, J. E. Van Cleve, J. D. Twicken, T. S. Barclay, M. N. Fanelli, F. R. Girouard, J. M. Jenkins, J. J. Kolodziejczak, Sean D. McCauliff, R. L. Morris, Kepler presearch data conditioning I — Architecture and algorithms for error correction in Kepler light curves. *Publ. Astron. Soc. Pac.* **124**, 985 (2012).

45. M. C. Stumpe, J. C. Smith, J. H. Catanzarite, J. E. Van Cleve, J. M. Jenkins, J. D. Twicken, F. R. Girouard, Multiscale systematic error correction via wavelet-based bandsplitting in *Kepler* data. *Publ. Astron. Soc. Pac.* **126**, 100 (2014).

46. J. D. Twicken, B. D. Clarke, S. T. Bryson, P. Tenenbaum, H. Wu, J. M. Jenkins, F. Girouard, T. C. Klaus, Photometric Analysis in the Kepler Science Operations Center Pipeline, in *Software and Cyberinfrastructure for Astronomy*, vol. 7740 of (2010), p. 774023.

47. R. L. Morris, J. D. Twicken, J. C. Smith, B. D. Clarke, J. M. Jenkins, S. T. Bryson, F. Girouard, T. C. Klaus, Kepler Data Processing Handbook: Photometric Analysis, Kepler Science Document KSCI-19081-003 (2020).

48. D. Rapetti, J. Jenkins, J. Twicken, D. Caldwell, Comparing and Automatically Optimizing the Performance of Systematic Error Correctors for TESS Light Curves, in *TESS Science Conference III* (2024), p. 17.

49. Lightkurve Collaboration, J. V. de M. Cardoso, C. Hedges, M. Gully-Santiago, N. Saunders, A. Cody, T. Barclay, O. Hall, S. Sagear, E. Turtelboom, J. Zhang, A. Tzanidakis, K. Mighell, J. Coughlin, K. Bell, Z. Berta-Thompson, P. Williams, J. Dotson, G. Barentsen, Lightkurve: Kepler and TESS time series analysis in Python, Astrophysics Source Code Library, record ascl:1812.013 (2018).

50. R. L. Gilliland, W. J. Chaplin, E. W. Dunham, V. S. Argabright, W. J. Borucki, G. Basri, S. T. Bryson, D. L. Buzasi, D. A. Caldwell, Y. P. Elsworth, J. M. Jenkins, D. G. Koch, J. Kolodziejczak, A. Miglio, J. van Cleve, L. M. Walkowicz, W. F. Welsh, *Kepler* mission stellar and instrument noise properties. *Astrophys. J. Suppl. Ser.* **197**, 6 (2011).

51. J. E. Van Cleve, S. B. Howell, J. C. Smith, B. D. Clarke, S. E. Thompson, S. T. Bryson, M. N. Lund, R. Handberg, W. J. Chaplin, That's how we roll: The NASA *K2* mission science products and their performance metrics. **128**, *Publ. Astron. Soc. Pac.* 075002 (2016).

52. H. P. Osborn, MonoTools: Planets of uncertain periods detector and modeler, Astrophysics Source Code Library, record ascl:2204.020 (2022).

53. H. P. Osborn, A. Bonfanti, D. Gandolfi, C. Hedges, A. Leleu, A. Fortier, D. Futyan, P. Gutermann, P. F. L. Maxted, L. Borsato, K. A. Collins, J. Gomes da Silva, Y. Gómez Maqueo Chew, M. J. Hooton, M. Lendl, H. Parviainen, S. Salmon, N. Schanche, L. M. Serrano, S. G. Sousa, A. Tuson, S. Ulmer-Moll, V. Van Grootel, R. D. Wells, T. G. Wilson, Y. Alibert, R. Alonso, G. Anglada, J. Asquier, D. Barrado y Navascues, W. Baumjohann, T. Beck, W. Benz, F. Biondi, X. Bonfils, F. Bouchy, A. Brandeker, C. Broeg, T. Bárczy, S. C. C. Barros, J. Cabrera, S. Charnoz, A. Collier Cameron, S. Csizmadia, M. B. Davies, M. Deleuil, L. Delrez, B.-O. Demory, D. Ehrenreich, A. Erikson, L. Fossati, M. Fridlund, M. Gillon, M. A. Gömez-Munoz, M. Güdel, K. Heng, S. Hoyer, K. G. Isaak, L. Kiss, J. Laskar, A. Lecavelier des Etangs, C. Lovis, D. Magrin, L. Malavolta, J. McCormac, V. Nascimbeni, G. Olofsson, R. Ottensamer, I. Pagano, E. Pallé, G. Peter, D. Piazza, G. Piotto, D. Pollacco, D. Queloz, R. Ragazzoni, N.

Rando, H. Rauer, C. Reimers, I. Ribas, O. D. S. Demangeon, A. M. S. Smith, L. Sabin, N. Santos, G. Scandariato, U. Schroffenegger, R. P. Schwarz, A. Shporer, A. E. Simon, M. Steller, G. M. Szabó, D. Ségransan, N. Thomas, S. Udry, I. Walter, N. Walton, Uncovering the true periods of the young sub-Neptunes orbiting TOI-2076. *Astron. Astrophys.* **664**, A156 (2022).

54. V. Van Eylen, S. Albrecht, Eccentricity from transit photometry: Small planets in *Kepler* multi-planet systems have low eccentricities. *Astrophys. J.* **808**, 126 (2015).

55. P. J. Wheatley, R. G. West, M. R. Goad, J. S. Jenkins, D. L. Pollacco, D. Queloz, H. Rauer, S. Udry, C. A. Watson, B. Chazelas, P. Eigmüller, G. Lambert, L. Genolet, J. M. Cormac, S. Walker, D. J. Armstrong, D. Bayliss, J. Bento, F. Bouchy, M. R. Burleigh, J. Cabrera, S. L. Casewell, A. Chaushev, P. Chote, S. Csizmadia, A. Erikson, F. Faedi, E. Foxell, B. T. Gänsicke, E. Gillen, A. Grange, M. N. Günther, S. T. Hodgkin, J. Jackman, A. Jordán, T. Louden, L. Metrailler, M. Moyano, L. D. Nielsen, H. P. Osborn, K. Poppenhaeger, R. Raddi, L. Raynard, A. M. S. Smith, M. Soto, R. Titz-Weider, The Next Generation Transit Survey (NGTS). *Mon. Not. R. Astron. Soc.* **475**, 4476–4493 (2018).

56. T. M. Brown, N. Baliber, F. B. Bianco, M. Bowman, B. Burleson, P. Conway, M. Crellin, É. Depagne, J. De Vera, B. Dilday, D. Dragomir, M. Dubberley, J. D. Eastman, M. Elphick, M. Falarski, S. Foale, M. Ford, B. J. Fulton, J. Garza, E. L. Gomez, M. Graham, R. Greene, B. Haldeman, E. Hawkins, B. Haworth, R. Haynes, M. Hidas, A. E. Hjelstrom, D. A. Howell, J. Hygelund, T. A. Lister, R. Lobdill, J. Martinez, D. S. Mullins, M. Norbury, J. Parrent, R. Paulson, D. L. Petry, A. Pickles, V. Posner, W. E. Rosing, R. Ross, D. J. Sand, E. S. Saunders, J. Shobbrook, A. Shporer, R. A. Street, D. Thomas, Y. Tsapras, J. R. Tufts, S. Valenti, K. V. Horst, Z. Walker, G. White, M. Willis, Las Cumbres Observatory Global Telescope network. **125**, *Publ. Astron. Soc. Pac.* 1031 (2013).

57. T. Guillot, L. Abe, A. Agabi, J. P. Rivet, J. B. Daban, D. Mékarnia, E. Aristidi, F. X. Schmider, N. Crouzet, I. Gonçalves, C. Gouvret, S. Ottogalli, H. Faradji, P. E. Blanc, E. Bondoux, F. Valbousquet, Thermalizing a telescope in Antarctica – Analysis of ASTEP observations. *Astron. Nachr.* **336**, 638–656 (2015).

58. D. Mékarnia, T. Guillot, J.-P. Rivet, F.-X. Schmider, L. Abe, I. Gonçalves, A. Agabi, N. Crouzet, T. Fruth, M. Barbieri, D. D. R. Bayliss, G. Zhou, E. Aristidi, J. Szulagyi, J.-B. Daban, Y. Fanteï-Caujolle, C. Gouvret, A. Erikson, H. Rauer, F. Bouchy, J. Gerakis, G. Bouchez, Transiting planet candidates with ASTEP 400 at Dome C, Antarctica. *Mon. Not. R. Astron. Soc.* **463**, 45–62 (2016).

59. C. McCully, N. H. Volgenau, D.-R. Harbeck, T. A. Lister, E. S. Saunders, M. L. Turner, R. J. Siverd, M. Bowman, Real-time processing of the imaging data from the network of Las Cumbres Observatory Telescopes using BANZAI, in, vol. 10707 of *Society of Photo-Optical Instrumentation Engineers (SPIE) Conference Series* (2018), p. 107070K.

60. K. A. Collins, J. F. Kielkopf, K. G. Stassun, F. V. Hessman, AstroImageJ: Image processing and photometric extraction for ultra-precise astronomical light curves. *Astron. J.* **153**, 77 (2017).

61. F. Marchis, A. Malvache, L. Marfisi, A. Borot, E. Arbouch, Unistellar eVscopes: Smart, portable, and easy-to-use telescopes for exploration, interactive learning, and citizen astronomy. *Acta Astronaut.* **166**, 23–28 (2020).

62. D. Queloz, M. Mayor, S. Udry, M. Burnet, F. Carrier, A. Eggenberger, D. Naef, N. Santos, F. Pepe, G. Rupprecht, G. Avila, F. Baeza, W. Benz, J.-L. Bertaux, F. Bouchy, C. Cavadore, B. Delabre, W. Eckert, J. Fischer, M. Fleury, A. Gilliotte, D. Goyak, J. C. Guzman, D. Kohler, D. Lacroix, J.-L. Lizon, D. Megevand, J.-P. Sivan, D. Sosnowska, U. Weilenmann, From CORALIE to HARPS. The way towards 1 m s^{-1} precision Doppler measurements. *Messenger* **105**, 1–7 (2001).

63. M. Mayor, F. Pepe, D. Queloz, F. Bouchy, G. Rupprecht, G. Lo Curto, G. Avila, W. Benz, J.-L. Bertaux, X. Bonfils, Th. Dall, H. Dekker, B. Delabre, W. Eckert, M. Fleury, A. Gilliotte, D. Gojak, J. C. Guzman, D. Kohler, J.-L. Lizon, A. Longinotti, C. Lovis, D. Megevand, L. Pasquini, J. Reyes, J.-P. Sivan, D. Sosnowska, R. Soto, S. Udry, A. van Kesteren, L. Weber, U. Weilenmann, Setting new standards with HARPS. *Messenger* **114**, 20–24 (2003).

64. J. D. Crane, S. A. Shectman, R. P. Butler, The Carnegie Planet Finder Spectrograph, in *Society of Photo-Optical Instrumentation Engineers (SPIE) Conference Series*, I. S. McLean, M. Iye,

Eds., vol. 6269 of *Society of Photo-Optical Instrumentation Engineers (SPIE) Conference Series* (2006), pp. 626931.

65. J. D. Crane, S. A. Shectman, R. P. Butler, I. B. Thompson, G. S. Burley, The Carnegie Planet Finder Spectrograph: A status report, in *Proceedings of the SPIE*, I. S. McLean, M. M. Casali, Eds. (2008), pp. 701479.

66. J. D. Crane, S. A. Shectman, R. P. Butler, I. B. Thompson, C. Birk, P. Jones, G. S. Burley, *The Carnegie Planet Finder Spectrograph: Integration and commissioning*, vol. 7735 of *Society of Photo-Optical Instrumentation Engineers (SPIE) Conference Series* (2010), pp. 773553.

67. R. P. Butler, G. W. Marcy, E. Williams, C. McCarthy, P. Dosanjh, S. S. Vogt, Attaining doppler precision of 3 M s^{-1}. *Publ. Astron. Soc. Pac.* **108**, 500 (1996).

68. A. Kaufer, O. Stahl, S. Tubbesing, P. Nørregaard, G. Avila, P. Francois, L. Pasquini, A. Pizzella, Commissioning FEROS, the new high-resolution spectrograph at La-Silla. *Messenger* **95**, 8–12 (1999).

69. B. Addison, D. J. Wright, R. A. Wittenmyer, J. Horner, M. W. Mengel, D. Johns, C. Marti, B. Nicholson, J. Soutter, B. Bowler, I. Crossfield, S. R. Kane, J. Kielkopf, P. Plavchan, C. G. Tinney, H. Zhang, J. T. Clark, M. Clerte, J. D. Eastman, J. Swift, M. Bottom, P. Muirhead, N. M. Crady, E. Herzig, K. Hogstrom, M. Wilson, D. Sliski, S. A. Johnson, J. T. Wright, J. A. Johnson, C. Blake, R. Riddle, B. Lin, M. Cornachione, T. R. Bedding, D. Stello, D. Huber, S. Marsden, B. D. Carter, Minerva-Australis. I. Design, commissioning, and first photometric results. *Publ. Astron. Soc. Pac.* **131**, 115003 (2019).

70. T. D. Morton, isochrones: Stellar model grid package. Astrophysics Source Code Library, record ascl:1503.010 (2015).

71. D. Foreman-Mackey, D. W. Hogg, D. Lang, J. Goodman, emcee: The MCMC Hammer. *Publ. Astron. Soc. Pac.* **125**, 306–312 (2013).

72. M. Zechmeister, M. Kürster, The generalised Lomb-Scargle periodogram. A new formalism for the floating-mean and Keplerian periodograms. *Astron. Astrophys.* **496**, 577–584 (2009).

73. S. Giacalone, C. D. Dressing, E. L. N. Jensen, K. A. Collins, G. R. Ricker, R. Vanderspek, S. Seager, J. N. Winn, J. M. Jenkins, T. Barclay, K. Barkaoui, C. Cadieux, D. Charbonneau, K. I. Collins, D. M. Conti, R. Doyon, P. Evans, M. Ghachoui, M. Gillon, N. M. Guerrero, R. Hart, E. Jehin, J. F. Kielkopf, B. M. Lean, F. Murgas, E. Palle, H. Parviainen, F. J. Pozuelos, H. M. Relles, A. Shporer, Q. Socia, C. Stockdale, T.-G. Tan, G. Torres, J. D. Twicken, W. C. Waalkes, I. A. Waite, Vetting of 384 TESS objects of interest with TRICERATOPS and statistical validation of 12 planet candidates. *Astron. J.* **161**, 24 (2021).

74. B. J. Fulton, E. A. Petigura, S. Blunt, E. Sinukoff, RadVel: The radial velocity modeling toolkit. *Publ. Astron. Soc. Pac.* **130**, 044504 (2018).

75. A. Venner, A. Vanderburg, L. A. Pearce, True masses of the long-period companions to HD 92987 and HD 221420 from Hipparcos-Gaia astrometry. *Astron. J.* **162**, 12 (2021).

76. A. Venner, Q. An, C. X. Huang, T. D. Brandt, R. A. Wittenmyer, A. Vanderburg, HD 28185 revisited: An outer planet, instead of a brown dwarf, on a Saturn-like orbit. *Mon. Not. R. Astron. Soc.* **535**, 90–106 (2024).

77. L. Kreidberg, batman: BAsic Transit Model cAlculatioN in Python. *Publ. Astron. Soc. Pac.* **127**, 1161–1165 (2015).

78. A. Venner, L. A. Pearce, A. Vanderburg, An edge-on orbit for the eccentric long-period planet HR 5183 b. *Mon. Not. R. Astron. Soc.* **516**, 3431–3446 (2022).

79. R. I. Dawson, J. A. Johnson, D. C. Fabrycky, D. Foreman-Mackey, R. A. Murray-Clay, L. A. Buchhave, P. A. Cargile, K. I. Clubb, B. J. Fulton, L. Hebb, A. W. Howard, D. Huber, A. Shporer, J. A. Valenti, Large eccentricity, low mutual inclination: The three-dimensional architecture of a hierarchical system of giant planets. *Astron. J.* **791**, 89 (2014).

80. J. M. Almenara, R. F. Díaz, G. Hébrard, R. Mardling, C. Damiani, A. Santerne, F. Bouchy, S. C. C. Barros, I. Boisse, X. Bonfils, A. S. Bonomo, B. Courcol, O. Demangeon, M. Deleuil, J. Rey, S. Udry, P. A. Wilson, SOPHIE velocimetry of *Kepler* transit candidates. XVIII. Radial

velocity confirmation, absolute masses and radii, and origin of the Kepler-419 multiplanetary system. *Astron. Astrophys.* **615**, A90 (2018).

81. K. Masuda, Eccentric companions to Kepler-448b and Kepler-693b: Clues to the formation of warm Jupiters. *Astron. J.* **154**, 64 (2017).

82. J. Korth, D. Gandolfi, J. Šubjak, S. Howard, S. Ataiee, K. A. Collins, S. N. Quinn, A. J. Mustill, T. Guillot, N. Lodieu, A. M. S. Smith, M. Esposito, F. Rodler, A. Muresan, L. Abe, S. H. Albrecht, A. Alqasim, K. Barkaoui, P. G. Beck, C. J. Burke, R. P. Butler, D. M. Conti, K. I. Collins, J. D. Crane, F. Dai, H. J. Deeg, P. Evans, S. Grziwa, A. P. Hatzes, T. Hirano, K. Horne, C. X. Huang, J. M. Jenkins, P. Kabáth, J. F. Kielkopf, E. Knudstrup, D. W. Latham, J. Livingston, R. Luque, S. Mathur, F. Murgas, H. L. M. Osborne, E. Palle, C. M. Persson, J. E. Rodriguez, M. Rose, P. Rowden, R. P. Schwarz, S. Seager, L. M. Serrano, L. Sha, S. A. Shectman, A. Shporer, G. Srdoc, C. Stockdale, T.-G. Tan, J. K. Teske, V. Van Eylen, A. Vanderburg, R. Vanderspek, S. X. Wang, J. N. Winn, TOI-1130: A photodynamical analysis of a hot Jupiter in resonance with an inner low-mass planet. *Astron. Astrophys.* **675**, A115 (2023).

83. J. Korth, P. Chaturvedi, H. Parviainen, I. Carleo, M. Endl, E. W. Guenther, G. Nowak, C. M. Persson, P. J. MacQueen, A. J. Mustill, J. Cabrera, W. D. Cochran, J. Lillo-Box, D. Hobbs, F. Murgas, M. Greklek-McKeon, H. Kellermann, G. Hébrard, A. Fukui, E. Pallé, J. M. Jenkins, J. D. Twicken, K. A. Collins, S. N. Quinn, J. Šubjak, P. G. Beck, D. Gandolfi, S. Mathur, H. J. Deeg, D. W. Latham, S. Albrecht, D. Barrado, I. Boisse, H. Bouy, X. Delfosse, O. Demangeon, R. A. García, A. P. Hatzes, N. Heidari, K. Ikuta, P. Kabáth, H. A. Knutson, J. Livingston, E. Martioli, M. Morales-Calderón, G. Morello, N. Narita, J. Orell-Miquel, H. L. M. Osborne, D. B. Palakkatharappil, V. Pinter, S. Redfield, H. M. Relles, R. P. Schwarz, S. Seager, A. Shporer, M. Skarka, G. Srdoc, M. Stangret, L. Thomas, V. Van Eylen, N. Watanabe, J. N. Winn, TOI-1408: Discovery and photodynamical modeling of a small inner companion to a hot Jupiter revealed by transit timing variations. *Astrophys. J. Lett.* **971**, L28 (2024).

84. H. Parviainen, R. Luque, E. Palle, Spright: A probabilistic mass–density–radius relation for small planets. *Mon. Not. R. Astron. Soc.* **527**, 5693–5716 (2023).

85. S. Hadden, Y. Lithwick, *Kepler* planet masses and eccentricities from TTV analysis. *Astron. J.* **154**, 5 (2017).

86. A. Leleu, J.-B. Delisle, L. Delrez, E. M. Bryant, A. Brandeker, H. P. Osborn, N. Hara, T. G. Wilson, N. Billot, M. Lendl, D. Ehrenreich, H. Chakraborty, M. N. Günther, M. J. Hooton, Y. Alibert, R. Alonso, D. R. Alves, D. R. Anderson, I. Apergis, D. Armstrong, T. Bárczy, D. Barrado Navascues, S. C. C. Barros, M. P. Battley, W. Baumjohann, D. Bayliss, T. Beck, W. Benz, L. Borsato, C. Broeg, M. R. Burleigh, S. L. Casewell, A. Collier Cameron, A. C. M. Correia, S. Csizmadia, P. E. Cubillos, M. B. Davies, M. Deleuil, A. Deline, O. D. S. Demangeon, B.-O. Demory, A. Derekas, B. Edwards, A. Erikson, A. Fortier, L. Fossati, M. Fridlund, D. Gandolfi, K. Gazeas, E. Gillen, M. Gillon, M. R. Goad, M. Güdel, F. Hawthorn, A. Heitzmann, C. Helling, K. G. Isaak, J. S. Jenkins, J. M. Jenkins, A. Kendall, L. L. Kiss, J. Korth, K. W. F. Lam, J. Laskar, D. W. Latham, A. Lecavelier des Etangs, D. Magrin, P. F. L. Maxted, J. McCormac, C. Mordasini, M. Moyano, V. Nascimbeni, G. Olofsson, A. Osborn, R. Ottensamer, I. Pagano, E. Pallé, G. Peter, G. Piotto, D. Pollacco, D. Queloz, R. Ragazzoni, N. Rando, H. Rauer, I. Ribas, G. Ricker, S. Saha, N. C. Santos, G. Scandariato, S. Seager, D. Ségransan, A. E. Simon, A. M. S. Smith, S. G. Sousa, M. Stalport, S. Sulis, G. M. Szabó, S. Udry, S. Ulmer-Moll, V. Van Grootel, R. Vanderspek, J. Venturini, E. Villaver, J. I. Vinés, N. A. Walton, R. G. West, P. J. Wheatley, J. Winn, T. Zivave, Photo-dynamical characterisation of the TOI-178 resonant chain. Exploring the robustness of transit-timing variations and radial velocity mass characterisations. *Astron. Astrophys.* **688**, A211 (2024).

87. H. Rein, D. Tamayo, G. Brown, High order symplectic integrators for planetary dynamics and their implementation in REBOUND *Mon. Not. R. Astron. Soc.* **489**, 4632–4640 (2019).

88. J. B. Irwin, The determination of a light-time orbit *Orbit* **116**, 211 (1952).

89. H. Parviainen, PYTRANSIT: Fast and easy exoplanet transit modelling in PYTHON. *Mon. Not. R. Astron. Soc.* **450**, 3233–3238 (2015).

90. H. Parviainen, J. Korth, Going back to basics: Accelerating exoplanet transit modelling using taylor-series expansion of the orbital motion. *Mon. Not. R. Astron. Soc.* **499**, 3356–3361 (2020).

91. H. Parviainen, RoadRunner: A fast and flexible exoplanet transit model. *Mon. Not. R. Astron. Soc.* **499**, 1633–1639 (2020).

92. R. Storn, K. Price, Differential evolution – A simple and efficient heuristic for global optimization over continuous spaces. *J. Glob. Optim.* **11**, 341–359 (1997).

93. K. Price, R. Storn, J. Lampinen, *Differential Evolution* (Springer, Berlin, 2005).

94. D. Foreman-Mackey, E. Agol, S. Ambikasaran, R. Angus, Fast and scalable gaussian process modeling with applications to astronomical time series. *Astron. J.* **154**, 220 (2017).

95. J. Wisdom, M. Holman, Symplectic maps for the n-body problem. *Astron. J.* **102**, 1528–1538 (1991).

96. C. D. Murray, S. F. Dermott, *Solar system dynamics* (Cambridge Univ. press, 2012).

97. C. Petrovich, E. Deibert, Y. Wu, Ultra-short-period planets from secular chaos. *Astron. J.* **157**, 180 (2019).

98. S. A. Baronett, N. Ferich, D. Tamayo, J. H. Steffen, Stellar evolution and tidal dissipation in REBOUNDx. *Mon. Not. R. Astron. Soc.* **510**, 6001–6009 (2022).

99. S. Millholland, G. Laughlin, Obliquity-driven sculpting of exoplanetary systems. *Nat. Astron.* **3**, 424–433 (2019).

100. D. Liveoak, S. C. Millholland, M. Vick, D. Tamayo, Self-consistent dynamical and chaotic tides in theREBOUNDxFramework. *Astrophys. J.* **989**, 35 (2025).

101. B. Levrard, C. Winisdoerffer, G. Chabrier, Falling transiting extrasolar giant planets. *Astrophys. J.* **692**, L9–L13 (2009).

102. N. P. Maffione, C. M. Giordano, P. M. Cincotta, Testing a fast dynamical indicator: The MEGNO. *Int. J. Non Linear Mech.* **46**, 23–34 (2011).

103. Y.-X. Chen, Z. Wang, Y.-P. Li, C. Baruteau, D. N. Lin, Wide dust gaps in protoplanetary disks induced by eccentric planets: A mass-eccentricity degeneracy. *Astrophys. J.* **922**, 184 (2021).

104. C. Baruteau, G. Wafflard-Fernandez, R. le Gal, F. Debras, A. Carmona, A. Fuente, P. Rivière-Marichalar, Observational signatures of eccentric Jupiters inside gas cavities in protoplanetary discs. *Mon. Not. R. Astron. Soc.* **505**, 359–376 (2021).

105. A. J. Mustill, M. B. Davies, A. Johansen, The destruction of inner planetary systems during high-eccentricity migration of gas giants. *Astrophys. J.* **808**, 14 (2015).

106. A. Socrates, B. Katz, S. Dong, S. Tremaine, Super-eccentric migrating Jupiters. *Astrophys. J.* **750**, 106 (2012).

107. A. J. Winter, G. P. Rosotti, C. Clarke, M. Giersz, Forming short-period substellar companions in 47 Tucanae–I. Dynamical model and brown dwarf tidal capture rates. *Mon. Not. R. Astron. Soc.* **509**, 3924–3937 (2021).

108. I. A. Bonnell, C. J. Clarke, M. R. Bate, M. J. McCaughrean, J. E. Pringle, H. Zinnecker, Are there brown dwarfs in globular clusters? *Mon. Not. R. Astron. Soc.* **343**, L53–L57 (2003).

109. J. Binney, S. Tremaine, *Galactic Dynamics: Second Edition* (Princeton Univ. Press, 2008).

110. H. Rein, D. M. Hernandez, D. Tamayo, G. Brown, E. Eckels, E. Holmes, M. Lau, R. Leblanc, A. Silburt, Hybrid symplectic integrators for planetary dynamics. *Mon. Not. R. Astron. Soc.* **485**, 5490–5497 (2019).

111. M. L. Wood, A. W. Mann, A. L. Kraus, Characterizing undetected stellar companions with combined data sets. *Astron. J.* **162**, 128 (2021).

112. A. H. M. J. Triaud, M. Neveu-VanMalle, M. Lendl, D. R. Anderson, A. C. Cameron, L. Delrez, A. Doyle, M. Gillon, C. Hellier, E. Jehin, P. F. L. Maxted, D. Ségransan, B. Smalley, D. Queloz, D. Pollacco, J. Southworth, J. Tregloan-Reed, S. Udry, R. West, Peculiar architectures for the WASP-53 and WASP-81 planet-hosting systems. *Mon. Not. R. Astron. Soc.* **467**, 1714–1733 (2017),.

113. N. Grieves, F. Bouchy, D. J. Armstrong, B. Akinsanmi, A. Psaridi, S. Ulmer-Moll, Y. G. C. Frensch, R. Helled, S. Müller, H. Knierim, N. C. Santos, V. Adibekyan, L. Parc, M. Lendl, M. P. Battley, N. Unger, G. Chaverot, D. Bayliss, X. Dumusque, F. Hawthorn, P. Figueira, Mar. A. F. Keniger, J. Lillo-Box, L. D. Nielsen, A. Osborn, S. G. Sousa, P. Strøm, S. Udry, Discovery of a cold giant planet and mass measurement of a hot super-Earth in the multi-planetary system WASP-132. *Astron. Astrophys.* **693**, A144 (2025).

114. I. Baraffe, G. Chabrier, F. Allard, P. H. Hauschildt, Evolutionary models for low-mass stars and brown dwarfs: Uncertainties and limits at very young ages. *Astron. Astrophys.* **382**, 563–572 (2002).

115. A. Burrows, K. Heng, T. Nampaisarn, The dependence of brown dwarf radii on atmospheric metallicity and Clouds: Theory and comparison with observations. *Astrophys. J.* **736**, 47 (2011).

116. G. Zhou, G. Á. Bakos, D. Bayliss, J. Bento, W. Bhatti, R. Brahm, Z. Csubry, N. Espinoza, J. D. Hartman, T. Henning, A. Jordán, L. Mancini, K. Penev, M. Rabus, P. Sarkis, V. Suc, M. Val-Borro, J. E. Rodriguez, D. Osip, L. Kedziora-Chudczer, J. Bailey, C. G. Tinney, S. Durkan, J. Lázár, I. Papp, P. Sári, HATS-70b: A 13 MJ brown dwarf transiting an A star *Astron. J.* **157**, 31 (2019).

117. A. Khandelwal, R. Sharma, A. Chakraborty, P. Chaturvedi, S. Ulmer-Moll, D. R. Ciardi, A. W. Boyle, S. Baliwal, A. Bieryla, D. W. Latham, N. J. S. S. V. Prasad, A. Nayak, M. Lendl, C. Mordasini, Discovery of a massive giant planet with extreme density around the sub-giant star TOI-4603. *Astron. Astrophys.* **672**, L7 (2023).

118. J. Šubjak, D. W. Latham, S. N. Quinn, P. Berlind, M. L. Calkins, G. A. Esquerdo, R. Brahm, J. A. Caballero, K. A. Collins, E. Guenther, J. Janík, P. Kabáth, R. P. Schwarz, T.-G. Tan, L. Vanzi, R. Zambelli, C. Ziegler, J. M. Jenkins, I. Mireles, S. Seager, A. Shporer, S. Striegel, J. N. Winn, Evolution of BD-14 3065b (TOI-4987b) from giant planet to brown dwarf as possible evidence of deuterium burning at old stellar ages. *Astron. Astrophys.* **688**, A120 (2024).

119. M. I. Jones, Y. Reinarz, R. Brahm, M. T. Pinto, J. Eberhardt, F. Rojas, A. H. M. J. Triaud, A. F. Gupta, C. Ziegler, M. J. Hobson, A. Jordán, T. Henning, T. Trifonov, M. Schlecker, N.

Espinoza, P. Torres-Miranda, P. Sarkis, S. Ulmer-Moll, M. Lendl, M. Uzundag, M. Moyano, K. Hesse, D. A. Caldwell, A. Shporer, M. B. Lund, J. M. Jenkins, S. Seager, J. N. Winn, G. R. Ricker, C. J. Burke, P. Figueira, A. Psaridi, K. A. Moulla, D. Mounzer, M. R. Standing, D. V. Martin, G. Dransfield, T. Baycroft, D. Dragomir, G. Boyle, V. Suc, A. W. Mann, M. Timmermans, E. Ducrot, M. J. Hooton, S. Zuñiga-Fernández, D. Sebastian, M. Gillon, D. Queloz, J. Carson, J. J. Lissauer, A long-period transiting substellar companion in the super-Jupiters to brown dwarfs mass regime and a prototypical warm-Jupiter detected by TESS. **683**, *Astron. Astrophys.* A192 (2024).

120. M. W. Phillips, P. Tremblin, I Baraffe, G. Chabrier, N. F. Allard, F. Spiegelman, J. M. Goyal, B. Drummond, E. Hébrard, A new set of atmosphere and evolution models for cool T-Y brown dwarfs and giant exoplanets. *Astron. Astrophys.* **637**, A38 (2020).

121. M. S. Marley, D. Saumon, C. Visscher, R. Lupu, R. Freedman, C. Morley, J. J. Fortney, C. Seay, A. J. R. W. Smith, D. J. Teal, R. Wang, The sonora brown dwarf atmosphere and evolution models. I. Model description and application to cloudless atmospheres in rainout chemical equilibrium. *Astrophys. J.* **920**, 85 (2021).

122. C. V. Morley, S. Mukherjee, M. S. Marley, J. J. Fortney, C. Visscher, R. Lupu, E. Gharib-Nezhad, D. Thorngren, R. Freedman, N. Batalha, The sonora substellar atmosphere models. III. Diamondback: Atmospheric properties, spectra, and evolution for warm cloudy substellar objects. *Astronphys. J.* **975**, 59 (2024).

123. I. Baraffe, G. Chabrier, T. S. Barman, F. Allard, P. H. Hauschildt, Evolutionary models for cool brown dwarfs and extrasolar giant planets. The case of HD 209458. *Astron. Astrophys.* **402**, 701–712 (2003).

124. I. Baraffe, D. Homeier, F. Allard, G. Chabrier, New evolutionary models for pre-main sequence and main sequence low-mass stars down to the hydrogen-burning limit. *Astron. Astrophys.* **577**, A42 (2015).

125. Z. Zhang, M. C. Liu, M. S. Marley, M. R. Line, W. M. J. Best, Uniform forward-modeling analysis of ultracool dwarfs. II. Atmospheric properties of 55 Late-T dwarfs. *Astrophys. J.* **921**, 95 (2021).

126. J. E. Owen, Y. Wu, *Kepler* planets: A tale of evaporation. *Astrophys. J.* **775**, 105 (2013).

127. L. A. Rogers, Most 1.6 Earth-radius planets are not rocky. *Astrophys. J.* **801**, 41 (2015).

128. B. J. Fulton, E. A. Petigura, The California-Kepler survey. VII. Precise planet radii leveraging Gaia DR2 reveal the stellar mass dependence of the planet radius gap. *Astrophys. J.* **156**, 264 (2018).

129. E. M. R. Kempton, J. L. Bean, D. R. Louie, D. Deming, D. D. B. Koll, M. Mansfield, J. L. Christiansen, M. López-Morales, M. R. Swain, R. T. Zellem, S. Ballard, T. Barclay, J. K. Barstow, N. E. Batalha, T. G. Beatty, Z. Berta-Thompson, J. Birkby, L. A. Buchhave, D. Charbonneau, N. B. Cowan, I. Crossfield, M. de Val-Borro, R. Doyon, D. Dragomir, E. Gaidos, K. Heng, R. Hu, S. R. Kane, L. Kreidberg, M. Mallonn, C. V. Morley, N. Narita, V. Nascimbeni, E. Pallé, E. V. Quintana, E. Rauscher, S. Seager, E. L. Shkolnik, D. K. Sing, A. Sozzetti, K. G. Stassun, J. A. Valenti, C. von Essen, A framework for prioritizing the TESS planetary candidates most amenable to atmospheric characterization. *Publ. Astron. Soc. Pac.* **130**, 114401 (2018).

130. A. D. Feinstein, R. A. Booth, J. B. Bergner, J. D. Lothringer, E. C. Matthews, L. Welbanks, Y. Miguel, B. Bitsch, L. E. J. Eriksson, J. Kirk, S. Pelletier, A. B. T. Penzlin, A. A. A. Piette, C. Piaulet-Ghorayeb, K. Schwarz, D. Turrini, L. Acuña-Aguirre, E.-M. Ahrer, M. G. Barber, J. Brande, A. Chakrabarty, I. J. M. Crossfield, G.-D. Marleau, H. Huang, A. Johansen, L. Kreidberg, J. H. Livingston, R. Luque, M. Oreshenko, E. Pacetti, G. Perotti, J. Polman, B. Prinoth, D. A. Semenov, J. B. Simon, J. Teske, N. Whiteford, On linking planet formation models, protoplanetary disk properties, and mature gas giant exoplanet atmospheres. arXiv:2506.00669 [astro-ph.EP] (2025).

131. Z. Essack, D. Dragomir, P. A. Dalba, M. P. Battley, D. R. Ciardi, K. A. Collins, S. B. Howell, M. I. Jones, S. R. Kane, E. E. Mamajek, C. R. Mann, I. Mireles, D. Oddo, L. A. Sgro, K. G. Stassun, S. Ulmer-Moll, C. N. Watkins, S. W. Yee, C. Ziegler, A. Bieryla, I. Apergis, K.

Barkaoui, R. Brahm, E. M. Bryant, T. M. Esposito, P. Figueira, B. J. Fulton, S. Gill, A. W. Howard, H. Isaacson, A. Kendall, N. Law, M. B. Lund, A. W. Mann, R. A. Matson, F. Murgas, E. Palle, S. N. Quinn, A. Revol, S. Saha, R. P. Schwarz, R. Sefako, A. Shporer, I. A. Strakhov, S. Villanueva Jr., G. R. Ricker, R. Vanderspek, D. W. Latham, S. Seager, J. N. Winn, P. Bosch-Cabot, K. I. Collins, R. Forés-Toribio, F. R. Frustagia, E. Girardin, I. J. Helm, P. Lewin, J. A. Muñoz, P. Newman, P. Plavchan, G. Srdoc, C. Stockdale, A. Wünsche, M. Billiani, M. Davy, A. Douvas, K. Fukui, B. Guillet, C. Ostrem, M. Rushton, A. Schmidt, A. Finardi, P. Girard, T. Goto, J. S. de Lambilly, L. Leroux, F. Mortecrette, J. W. Pickering, M. Primm, M. Ribot, E. Teng, A. Verveen, S. Will, M. Ziegler, Giant outer transiting exoplanet mass (GOT 'EM) Survey. VI. Confirmation of a long-period giant planet discovered with a single TESS transit. *Astron. J.* **170**, 41 (2025).

132. B. Ma, J. Ge, Statistical properties of brown dwarf companions: Implications for different formation mechanisms. *Mon. Not. R. Astron. Soc.***439**, 2781–2789 (2014).

133. K. C. Schlaufman, Evidence of an upper bound on the masses of planets and its implications for giant planet formation. *Astrophys. J.* **853**, 37 (2018).

134. J. Maldonado, E. Villaver, Searching for chemical signatures of brown dwarf formation. *Astron. Astrophys.* **602**, A38 (2017).

135. B. A. Henderson, S. L. Casewell, A. Jordán, R. Brahm, T. Henning, S. Gill, L C Mayorga, C. Ziegler, K. G. Stassun, M. R. Goad, J. Acton, D. R. Alves, D. R. Anderson, I. Apergis, D. J. Armstrong, D. Bayliss, M. R. Burleigh, D. Dragomir, E. Gillen, M. N. Günther, C. Hedges, K. M. Hesse, M. J. Hobson, J. S. Jenkins, J. M. Jenkins, A. Kendall, M. Lendl, M. B. Lund, J. M. Cormac, M. Moyano, A. Osborn, M. T. Pinto, G. Ramsay, D. Rapetti, S. Saha, S. Seager, T. Trifonov, S. Udry, J. I. Vines, R. G. West, P. J. Wheatley, J. N. Winn, T. Zivave, TOI-2490b – The most eccentric brown dwarf transiting in the brown dwarf desert. *Mon. Not. R. Astron. Soc.* **533**, 2823–2842 (2024).

136. K. G. Stassun, R. J. Oelkers, J. Pepper, M. Paegert, N. De Lee, G. Torres, D. W. Latham, S. Charpinet, C. D. Dressing, D. Huber, S. R. Kane, S. Lépine, A. Mann, P. S. Muirhead, B. Rojas-

Ayala, R. Silvotti, S. W. Fleming, A. Levine, P. Plavchan, The TESS input catalog and candidate target list. *Astron. J.* **156**, 102 (2018).

137. Gaia Collaboration, Gaia Data Release 3. Summary of the content and survey properties. *Astron. Astrophys.* **674**, A1 (2023).

138. A. A. Henden, APASS DR10 Has Arrived! (Abstract). *J. Am. Assoc. Var. Star Observ.* **47**, 130 (2019).

139. R. M. Cutri, M. F. Skrutskie, S. van Dyk, C. A. Beichman, J. M. Carpenter, T. Chester, L. Cambresy, T. Evans, J. Fowler, J. Gizis, E. Howard, J. Huchra, T. Jarrett, E. L. Kopan, J. D. Kirkpatrick, R. M. Light, K. A. Marsh, H. McCallon, S. Schneider, R. Stiening, M. Sykes, M. Weinberg, W. A. Wheaton, S. Wheelock, N. Zacarias, VizieR Online Data Catalog: 2MASS All-Sky Catalog of Point Sources (Cutri+ 2003), VizieR On-line Data Catalog: II/246. Originally published in: University of Massachusetts and Infrared Processing and Analysis Center, (IPAC/California Institute of Technology) (2003) (2003).

140. A. Reuther, J. Kepner, C. Byun, S. Samsi, W. Arcand, D. Bestor, B. Bergeron, V. Gadepally, M. Houle, M. Hubbell, M. Jones, A. Klein, L. Milechin, J. Mullen, A. Prout, A. Rosa, C. Yee, P. Michaleas, Interactive supercomputing on 40,000 cores for machine learning and data analysis, in *2018 IEEE High Performance extreme Computing Conference (HPEC)* (IEEE, 2018), pp. 1–6.
